# Plasticizer-Dependent Vulcanization, Crosslinking, and Magneto-Viscoelasticity of Natural Rubber-Based Magnetorheological Elastomers

**DOI:** 10.3390/polym18182195

**Published:** 2026-09-09

**Authors:** Lili Fan, Haimin Zhu, Jianhua Du, Zhichao Li, Haisong Wu, Yicheng Su, Wangwei Li, Junhui Yao

**Affiliations:** 1 School of Automotive Engineering, Suzhou University of Technology, Suzhou 215000, China; 2 School of Automobile and Traffic Engineering, Liaoning University of Technology, Jinzhou 121001, China17861192018@163.com (W.L.)

**Keywords:** magnetorheological elastomers, natural rubber, plasticizers, vulcanization kinetics, crosslinking state, magneto-viscoelasticity

## Abstract

Adaptive vibration control under variable dynamic loading requires damping materials that combine structural stability, efficient energy dissipation, and field-tunable mechanical response. Here, natural-rubber-based magnetorheological elastomers containing coumarone resin (MRE-C), naphthenic oil (MRE-N), or paraffin (MRE-P) were systematically compared through microstructural characterization, dynamic viscoelastic testing, vulcanization kinetics, and equilibrium swelling. MRE-C exhibited pronounced strain-induced softening, whereas MRE-P showed a high relative dynamic sensitivity but limited absolute stiffness. MRE-N achieved the most favorable overall balance, with more continuous anisotropic particle-chain structures and the lowest Payne-effect amplitude (37.90%). At 5 A, its storage modulus increased by 25.8%, close to the 30.9% increase in MRE-P, while maintaining substantially higher absolute G′ and G″ and a nearly unchanged tan δ. MRE-N’s macroscopic advantage was associated with more favorable network formation, with 17.9% and 7.4% lower apparent activation energies and 7.2% and 18.1% higher apparent crosslink densities than MRE-C and MRE-P, respectively. Molecular simulations of representative sulfur bridges further indicated that monosulfidic bridges favored geometric constraint and structural recovery, whereas disulfidic bridges exhibited greater conformational adaptability. These results link plasticizer-dependent vulcanization and crosslinking state with macroscopic magneto-viscoelastic performance and identify naphthenic oil as the most effective of the investigated plasticizers for balancing stiffness, energy dissipation, and magnetic responsiveness.

## 1. Introduction

Engineering structures in wind-energy, offshore, transportation, and other vibration-sensitive applications are frequently subjected to variable dynamic disturbances, including vibration, cyclic deformation, and impact [1,2,3,4,5]. Because the fixed mechanical properties of conventional passive damping materials can limit their adaptability to changing operating conditions, materials combining mechanical stability with controllable energy dissipation are highly desirable.

Magnetorheological elastomers (MREs) are magnetic-field-responsive composites consisting of magnetizable particles embedded in a crosslinked elastomer matrix. Under an applied magnetic field, interactions among the particles produce reversible changes in stiffness and damping, enabling tunable viscoelastic behavior and making MREs attractive for adaptive vibration isolation, vibration absorption, and semi-active vibration control [6,7,8,9,10]. However, the overall magneto-viscoelastic performance of MREs depends not only on the magnetic particles but also on the mechanical state of the elastomer matrix. For natural-rubber-based MREs, sulfur vulcanization establishes the crosslinked network that largely determines matrix elasticity, deformation stability, and viscoelastic dissipation, while crosslink density and network structure are closely associated with the mechanical response under cyclic deformation [11,12,13,14]. Accordingly, the vulcanization and crosslinking state of the matrix constitutes an important structural basis for understanding the magneto-viscoelastic behavior of natural-rubber-based MREs.

Plasticizers are important formulation variables because their molecular characteristics and compatibility with the rubber matrix can affect vulcanization, matrix mobility, and dynamic mechanical behavior [15,16]. Beyond conventional petroleum-based processing oils, alternative plasticizers such as castor oil, cardanol, and palm oil have also been investigated in NR compounds. Comparative studies have reported differences in cure behavior, crosslinking characteristics, and mechanical and dynamic properties compared with naphthenic or aromatic oils [17,18,19]. In NR-based MREs, plasticizer type has also been shown to influence curing characteristics and field-dependent rheological response [20,21]. Nevertheless, how plasticizer-dependent vulcanization and crosslinking state are related to the balance among mechanical stability, energy dissipation, and magnetorheological response remains insufficiently understood. Clarifying this relationship is essential for identifying plasticizer formulations that provide favorable overall MRE performance rather than maximizing a single response parameter.

In this study, coumarone resin, naphthenic oil, and paraffin were selected as plasticizers with distinct physicochemical characteristics to establish a controlled comparative system. We investigate how plasticizer-induced differences in vulcanization and crosslinking state are associated with the balance among mechanical stability, energy dissipation, and magnetic-field responsiveness in natural-rubber-based MREs. Three plasticizer formulations were systematically compared to identify the formulation with the most favorable overall performance. The selected MRE was then further analyzed using an established vulcanization kinetic model to evaluate the competition between crosslink formation and network degradation. Representative mono- and disulfidic bridges were subsequently examined through molecular simulations to assess their structural responses under thermal perturbations. This stepwise analysis links plasticizer-dependent vulcanization with macroscopic magneto-viscoelastic performance and further examines crosslink-network stability through representative sulfur-bridge structures.

## 2. Experimental and Computational Methods

### 2.1. Materials and Fabrication of MREs

The raw materials used for preparing magnetorheological elastomers (MREs) included an elastomeric matrix, a magneto-responsive filler, plasticizers, vulcanization additives, and antioxidants. Ribbed smoked sheet natural rubber (RSS1#, Palembang, Indonesia), purchased from Nanjing Jinsanli Rubber and Plastic Co., Ltd. (Nanjing, China), was used as the elastomeric matrix. Carbonyl iron powder (CIP, grade EW, 3–4 μm, BASF SE, Ludwigshafen, Germany) was employed as the magneto-responsive filler. Coumarone resin was purchased from Jinan Guofeng Chemical Co., Ltd. (Jinan, China), whereas naphthenic oil and paraffin were supplied by Wuhu Huzhan Rubber Chemical Co., Ltd. (Wuhu, China). Zinc oxide (ZnO) and stearic acid (SA), used as the vulcanization activator system, were obtained from Chengdu Kelong Chemical Reagent Factory (Chengdu, China). Accelerator CZ, antioxidant RD, and antioxidant 4010NA were supplied by Guangdong Duba New Material Technology Co., Ltd. (Dongguan, China). Their full chemical names and chemical structures are summarized in Table 1. Sulfur was purchased from Nanjing Ningcheng Chemical Reagent Co., Ltd. (Nanjing, China). All materials were used as received without further purification.

During formulation design, three MRE formulations were prepared by changing only the plasticizer type while maintaining the same magnetic filler loading and sulfur vulcanization system. The CIP content was fixed at approximately 58.91 wt% (190 phr) of the total compound to provide a high magnetic-particle loading and ensure stable magneto-responsive behavior. Coumarone resin, naphthenic oil, and paraffin were introduced as plasticizers at the same mass-based loading of approximately 3.72 wt% (12 phr), thereby maintaining a constant plasticizer-to-rubber ratio for a controlled comparison of plasticizer type. The curing-related components were kept constant among the three formulations. ZnO and SA accounted for approximately 1.55 wt% and 0.31 wt% (5 and 1 phr), respectively; Accelerator CZ accounted for approximately 0.16 wt% (0.5 phr), sulfur accounted for approximately 0.93 wt% (3 phr); and antioxidants RD and 4010NA accounted for approximately 0.93 wt% and 0.62 wt% (3 and 2 phr), respectively. A conventional sulfur vulcanization system was adopted for all MRE formulations to ensure that the effect of plasticizer type could be compared under the same curing background. In this curing system, sulfur acted as the vulcanizing agent, accelerator CZ promoted sulfur vulcanization, and ZnO/SA served as the activator system. The sulfur and accelerator CZ contents were approximately 0.93 wt% and 0.16 wt%, respectively, corresponding to a sulfur-to-accelerator mass ratio of about 5.8:1. This relatively high sulfur-to-accelerator ratio is characteristic of a conventional sulfur vulcanization system, which tends to generate sulfur-rich crosslinked networks containing flexible polysulfidic linkages. Such crosslink structures are beneficial for elastic recovery and dynamic deformation tolerance, whereas their stability is sensitive to plasticizer-dependent chain mobility, additive diffusion, and curing efficiency. Therefore, maintaining the same vulcanization system provides a consistent basis for comparing plasticizer-mediated sulfur-crosslinked network stability and dynamic energy dissipation behavior.

**Figure 1:**
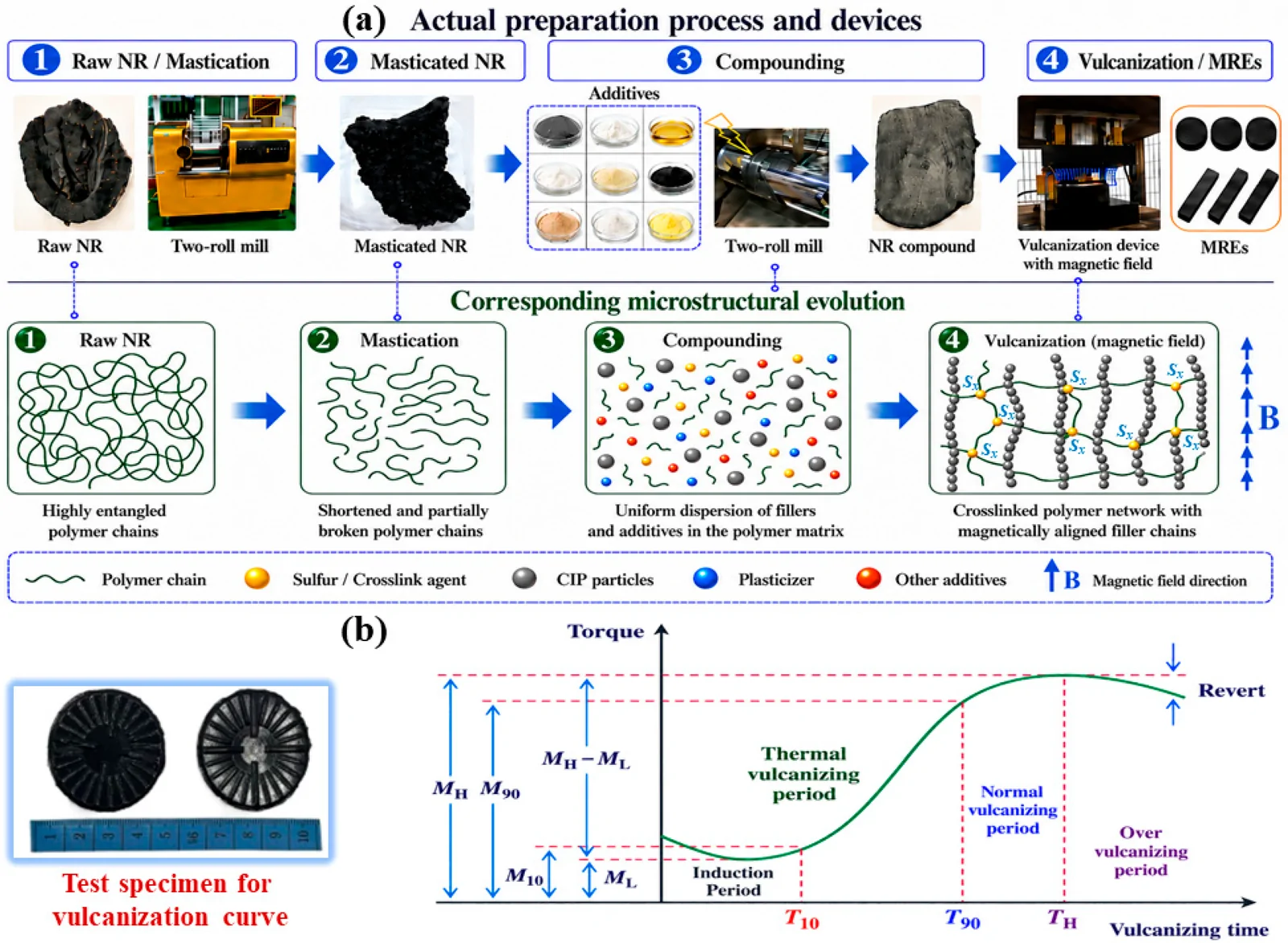
(**a**) Fabrication of MREs. (**b**) Vulcanization curve of MREs.

The MREs were fabricated through mastication, compounding, and magnetic-field-assisted vulcanization, as schematically illustrated in Figure 1a. The raw NR was first masticated on a two-roll mill (LN-6, Guangdong Lina Enterprise Co., Ltd., Dongguan, China), with the front and rear rollers maintained at 55 °C and 50 °C, respectively. Mechanical shear during mastication reduced molecular chain entanglement and improved the processability of the NR matrix. Subsequently, CIP, the selected plasticizer, ZnO, SA, accelerator CZ, antioxidants, and sulfur were incorporated into the masticated NR according to the designed formulation. The compounding process was continued for 25 min to promote homogeneous dispersion of CIP and uniform distribution of the vulcanization additives within the rubber matrix. After compounding, the uncured rubber compound was transferred into the mold cavity and vulcanized using a self-designed magnetic-field-assisted curing apparatus. The vulcanization time of each formulation was determined according to the optimum curing time measured by a rotorless rheometer (CRS-VTM200, Suzhou Yanuo Tianxia Instrument Co., Ltd., Suzhou, China). During vulcanization, sulfur crosslinking reactions convert the NR matrix into a three-dimensional elastic network. Meanwhile, CIPs are magnetized under the applied magnetic field and assembled into chain-like or cluster-like structures along the field direction. As the sulfur-crosslinked network developed, these field-induced particle assemblies are immobilized within the NR matrix, yielding different plasticizer-dependent anisotropic MREs, denoted as MRE-C (Coumarone resin), MRE-N (naphthenic oil), and MRE-P (Paraffin), respectively. After vulcanization, the samples were demolded, cooled to room temperature, and sectioned into cylindrical and rectangular specimens matching the dimensions required by the testing apparatus for subsequent dynamic energy dissipation behavior analysis.

This fabrication process couples plasticizer-dependent chain mobility, magnetic particle organization, and sulfur-crosslinked network formation. Mastication and compounding provide a processable rubber matrix for additive diffusion and CIP rearrangement, whereas magnetic-field-assisted vulcanization fixes the field-induced particle assemblies through the crosslinked NR network. The resulting microstructure and sulfur-crosslinked network stability are expected to govern the dynamic mechanical response and energy dissipation behavior of the MREs.

### 2.2. Characterization of MREs

The microstructure, dynamic mechanical properties, and vulcanization behavior of the magnetorheological elastomers (MREs) were systematically characterized using scanning electron microscopy (SEM, COXEM EM-CX, Daejeon, Republic of Korea), a rotational rheometer (MCR 302, Anton Paar, Graz, Austria), and a rotorless vulcanization rheometer (CRS-VTM200, Yangzhou Yuanfeng Testing Equipment Co., Ltd., Yangzhou, China). SEM was employed to visualize the internal morphology and magnetic particle distribution within the sulfur-crosslinked network. Prior to observation, samples were cryogenically fractured in liquid nitrogen and sputter-coated with a thin gold layer to eliminate charging effects under electron beam irradiation. The rotational rheometer was operated in oscillatory shear mode to quantify the viscoelastic response, including storage modulus (G′), loss modulus (G″), and damping factor (tan δ), thereby revealing the energy storage and dissipation characteristics under dynamic deformation. The rotorless rheometer records the torque evolution of a rubber specimen subjected to small-amplitude oscillatory deformation in a sealed cavity, enabling real-time monitoring of sulfur vulcanization kinetics and crosslink network development. The crosslink density of the MREs was determined by equilibrium swelling, with the detailed measurement procedure and calculation provided in Section 3.3.2.

The vulcanization characteristics were evaluated using the vulcanization curve, as shown in Figure 1b, where the torque response as a function of time reflects the evolution of the sulfur-crosslinked network. The vulcanization process typically consists of four stages, namely, induction period, curing acceleration stage, optimal curing plateau, and reversion stage. The minimum torque (ML) and maximum torque (MH) represent the uncured and fully developed crosslinking states, respectively. Key parameters including scorch time (*T*_10_), optimum curing time (*T*_90_), and curing rate index (*V*_c_) were extracted to quantify curing kinetics. The curing rate is defined as $V_{c}=100/(T_{90}-T_{s1})$, where *T*_S1_ denotes the scorch time, i.e., the duration of the induction period. The difference (MH − ML) is used as an indirect indicator of crosslink density and network formation.

### 2.3. Molecular Modeling and Simulation Method

**Figure 2:**
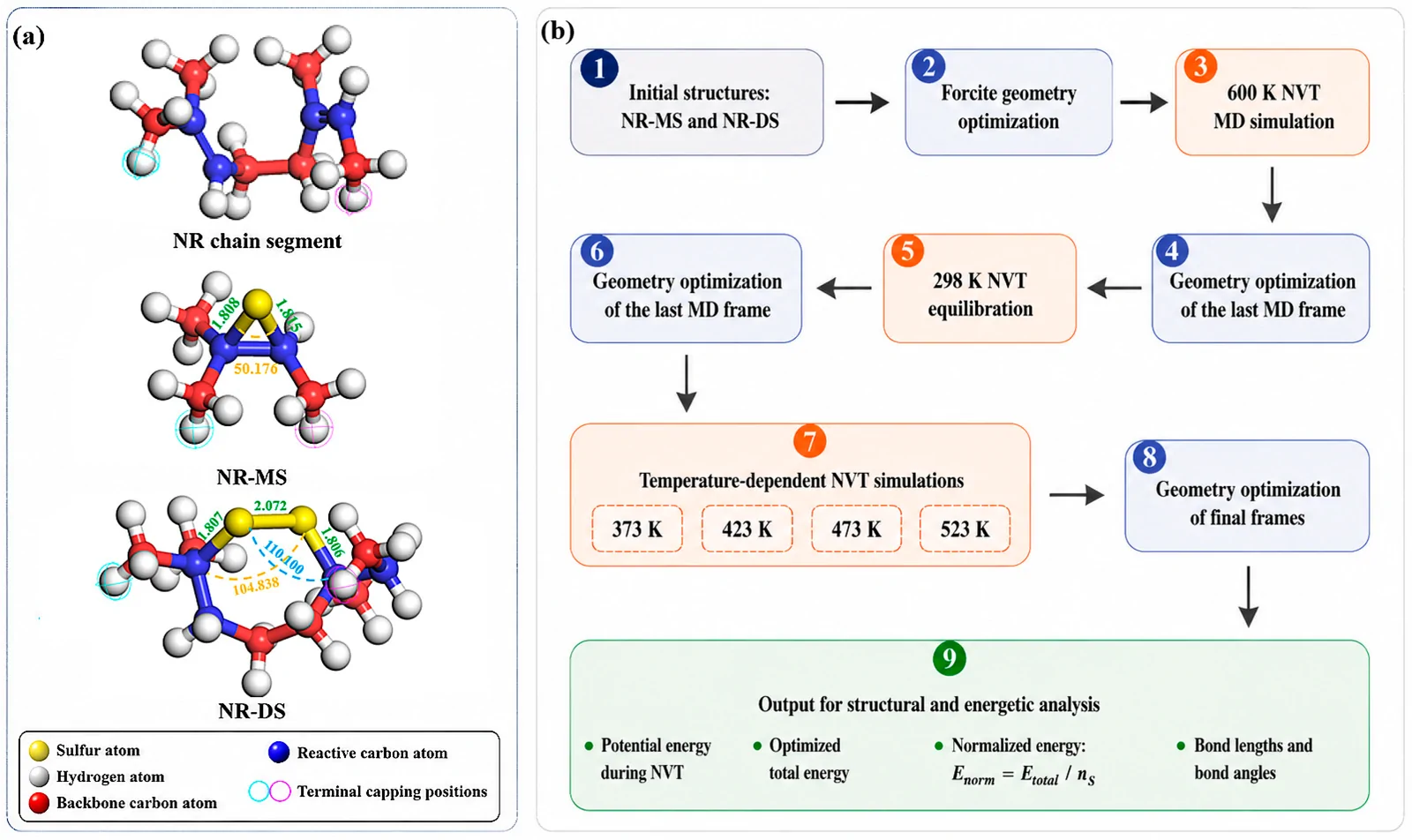
(**a**) NR chain segment and sulfur-crosslinked structures. (**b**) Molecular simulation workflow.

Molecular modeling and simulation were conducted to investigate the local structural configurations of sulfur-crosslinked natural rubber, as illustrated in Figure 2. A cis-1,4-polyisoprene segment was employed as the representative molecular backbone of natural rubber (NR), from which two typical sulfur-crosslinked configurations were constructed.

As shown in Figure 2a, the pristine NR chain segment was defined as the reference structure. Based on this backbone, two representative local vulcanization models were constructed by introducing sulfur atoms at the reactive carbon sites originally associated with the C=C double bond. The resulting structures include a monosulfidic bridge model (NR-MS) and a disulfidic bridge model (NR-DS). In the NR-MS structure, a single sulfur atom bridges two carbon atoms to form a localized C–S–C cyclic configuration. In contrast, the NR-DS structure contains two consecutive sulfur atoms connecting the same carbon sites, forming a C–S–S–C chain-like linkage. Both models were constructed with identical backbone length and topology, and hydrogen atoms were used to cap terminal chain ends to eliminate boundary effects, ensuring consistent structural comparison.

All calculations were performed using the Forcite module in Materials Studio 2020. The COMPASS III force field was applied throughout all simulations. Van der Waals interactions were evaluated using the atom-based summation method with a cutoff distance of 15.5 Å, and atomic charges were assigned using the forcefield-assigned scheme. As illustrated in Figure 2b, an identical computational protocol was applied to the NR-MS and NR-DS models and comprised two interconnected procedures—structural optimization and thermal perturbation. Geometry optimization was performed before and after each molecular dynamics (MD) stage to obtain energetically relaxed configurations, whereas thermal perturbation was introduced through canonical ensemble (NVT) simulations to evaluate the temperature-dependent structural and energetic responses of the two models. The initial structures were first optimized using the Forcite module and subsequently subjected to NVT simulations at 600 K to enhance conformational sampling and facilitate escape from local energy minima. The final frame of each high-temperature trajectory was then extracted and re-optimized to eliminate thermally induced distortions. The resulting structures were equilibrated under the NVT ensemble at 298 K, followed by further geometry optimization of the corresponding final frames. These equilibrated and optimized configurations were subsequently used as the starting structures for independent NVT simulations at 373, 423, 473, and 523 K. The final frame obtained at each temperature was further optimized to generate fully relaxed configurations for structural and energetic characterization. Representative bond lengths and bond angles were extracted from the optimized structures to quantify local structural changes induced by temperature. The trajectory-averaged potential energy was used to characterize the dynamic energetic response during the NVT simulations, whereas the total energy of each optimized configuration was employed to assess the stability of the thermally perturbed structures. To eliminate the influence of different sulfur atom numbers between the two models, the optimized total energy obtained from Forcite calculations was further normalized according to Equation (1).

(1) $$E_{\mathrm{norm}}=E_{\mathrm{total}}/n_{S}$$

where *E*_norm_ represents the sulfur-atom-normalized energy; *E*_total_ denotes the total energy after geometry optimization; and *n*_S_ is the number of sulfur atoms in each model, with values of 1 and 2 for NR-MS and NR-DS, respectively. This unified computational workflow enables a consistent and systematic comparison of the conformational relaxation, thermal response, and energetic stability of the NR-MS and NR-DS models.

## 3. Results and Discussion

### 3.1. Plasticizer-Dependent Microstructural Characteristics of MREs

**Figure 3:**
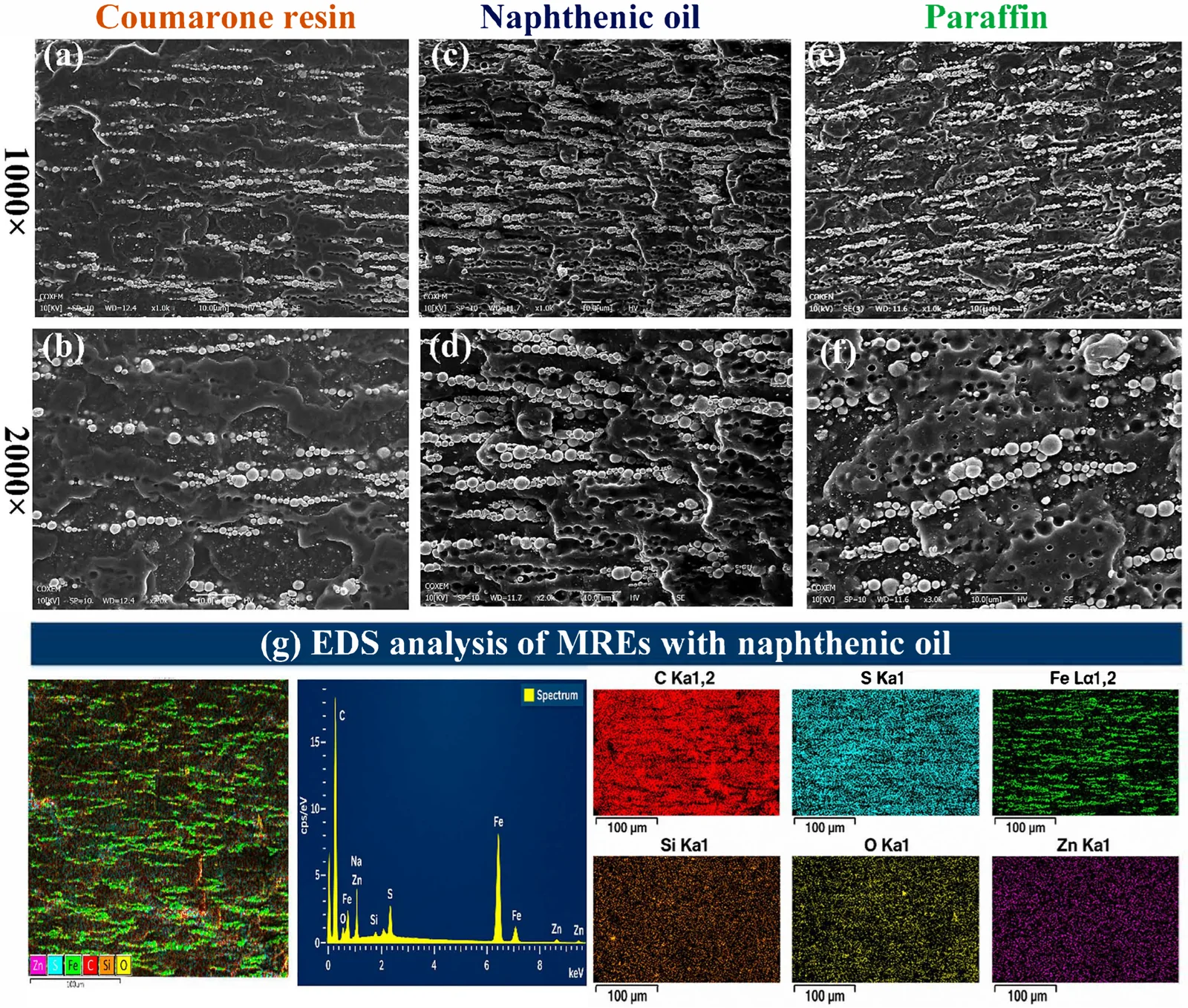
Plasticizer-dependent microstructure and elemental mapping of MREs. (**a**) coumarone resin, 1000×; (**b**) coumarone resin, 2000×; (**c**) naphthenic oil, 1000×; (**d**) naphthenic oil, 2000×; (**e**) paraffin, 1000×; (**f**) paraffin, 2000×; (**g**) EDS analysis of MREs with naphthenic oil.

Figure 3 presents the plasticizer-dependent microstructural evolution of magnetorheological elastomers (MREs), where coumarone resin, naphthenic oil, and paraffin were employed as the plasticizers. The SEM images at different magnifications (1000× and 2000×) reveal pronounced differences in the dispersion state of carbonyl iron particles (CIPs) and the formation of field-induced chain-like architectures within the natural rubber (NR) matrix.

For the coumarone resin-based MREs (Figure 3a,b), the microstructure exhibits relatively constrained particle mobility, leading to discontinuous and locally aggregated CIP distributions. The formation of chain-like structures is limited, and particle alignment along the field direction is weak, indicating restricted matrix-assisted rearrangement during curing. In contrast, the naphthenic oil-plasticized system (Figure 3c,d) shows the most pronounced and continuous chain-like architectures. CIPs are highly aligned into well-defined linear assemblies with improved interparticle connectivity. This behavior suggests that naphthenic oil provides an optimal balance between polymer chain mobility and particle dispersion, effectively facilitating magnetic-field-driven particle migration and alignment during the curing process. For the paraffin-based system (Figure 3e,f), although local particle aggregation is observed, the chain continuity is partially disrupted and the spatial distribution becomes less uniform. This indicates reduced compatibility between paraffin and the rubber matrix, which limits particle reorganization and weakens field-induced structuring efficiency. Taken together, these observations suggest that the different particle-alignment states arise from the distinct effects of the plasticizers on matrix mobility and the ability of CIPs to rearrange during curing. Such a mechanism is consistent with previous studies showing that the type of plasticizing medium can influence particle dispersion, mobility, and microstructural development in MREs [20,22]. Naphthenic oil appears to provide a more favorable environment for field-induced particle migration and alignment before the rubber network is fixed by vulcanization, whereas coumarone resin and paraffin impose relatively greater constraints on particle rearrangement, resulting in less continuous chain structures.

To further elucidate the elemental origin of the observed microstructures, energy-dispersive X-ray spectroscopy (EDS) was performed on the naphthenic oil-based MREs (Figure 3g). The spectrum confirms the presence of C, Fe, S, Si, O, and Zn as the main elemental constituents. Fe and Si are primarily associated with carbonyl iron particles (CIPs), while C and O originate from the rubber matrix and plasticizer phase. S and Zn are attributed to the vulcanization system and additives. In addition, Elemental mapping further reveals that Fe-rich regions exhibit pronounced chain-like alignment consistent with SEM-observed structures, confirming that the magnetic particles are responsible for the anisotropic microstructural features. In contrast, C, S, O, Si, and Zn are uniformly distributed throughout the matrix, indicating their homogeneous incorporation into the crosslinked rubber network rather than participation in particle aggregation.

Overall, the combined SEM and EDS results confirm that plasticizer type plays a key role in regulating CIP rearrangement and the resulting anisotropic microstructure of MREs. Among the three systems, naphthenic oil most effectively promotes the formation of continuous Fe-rich particle chains, resulting in the most pronounced field-induced anisotropy.

### 3.2. Plasticizer-Dependent Viscoelastic Response

The magneto-viscoelastic behavior of MREs was systematically investigated under coupled mechanical and magnetic loading conditions, including precompression, strain amplitude, frequency, and applied magnetic field. These measurements were conducted to elucidate how plasticizer-dependent polymer chain mobility, magnetic particle rearrangement, and network confinement synergistically determine the mechanical stability, magneto-responsive performance of MREs, and energy dissipation behavior.

#### 3.2.1. Precompression Effect on Magneto-Mechanical Stability of MREs

**Figure 4:**
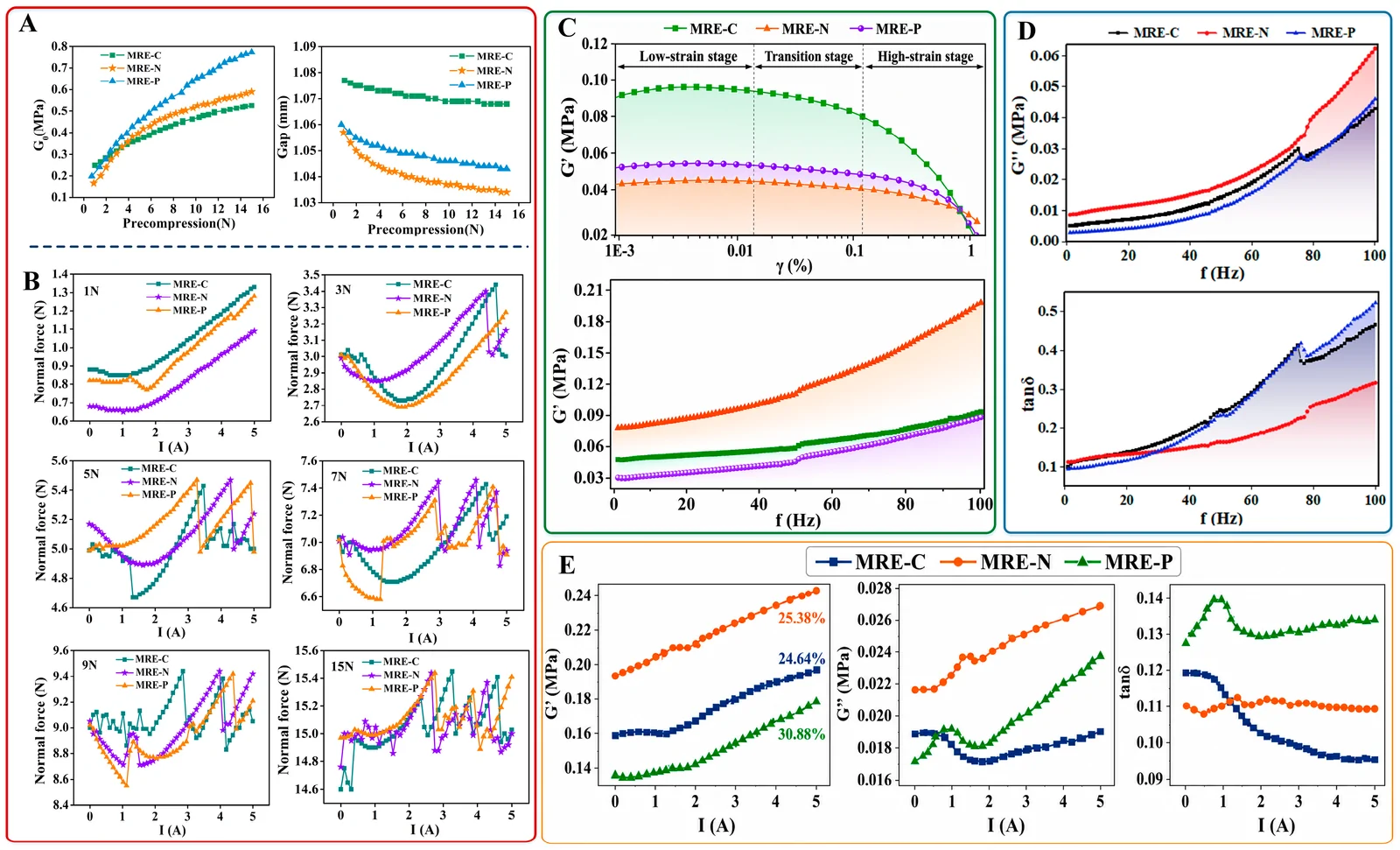
Magneto-viscoelastic behavior and energy dissipation of MREs under coupled loading. (**A**) Effect of precompression on the zero-field modulus and gap of MREs. (**B**) Precompression effect on the normal force of MREs. (**C**) Strain- and frequency-dependent storage modulus. (**D**) Frequency-dependent loss modulus and damping. (**E**) Magnetic-field-dependent viscoelasticity.

In practical applications, MREs are frequently subjected to external precompression, which can alter the packing state of magnetic particles and consequently affect their magneto-mechanical response. Therefore, the influence of precompression on the mechanical reinforcement and field-induced response stability of different plasticizer-dependent MREs was systematically investigated. As shown in Figure 4A (Left), the storage modulus (G′) of all MREs increases continuously with increasing precompression, indicating that compressive loading enhances particle confinement and strengthens internal interactions within the particle-filled network. When the precompression increases to 15 N, the storage modulus of MRE-C, MRE-N, and MRE-P increases by approximately 1.12, 2.54, and 2.89 times, respectively. This enhancement can be phenomenologically interpreted using a Mooney-type particle-crowding relation [23], as shown in Equation (2).

(2) $$G_{0}=G_{p}\exp\left(\frac{2.5\varphi }{1-S\varphi }\right)$$

where $G_{0}$ represents the zero-field shear modulus of the MRE, $G_{p}$ denotes the intrinsic shear modulus of the polymer matrix, φ is the volume fraction of magnetic particles, and S is the crowding factor associated with particle–particle interactions and packing effects.

According to the expression, increasing precompression reduces the interparticle distance and increases particle packing density, thereby strengthening particle–particle interactions and improving the zero-field modulus of the composite. This mechanism is further supported by the precompression-dependent gap evolution of the MREs shown in Figure 4A (Right), with the gap automatically monitored and recorded by a rotational rheometer during loading. With increasing precompression, the gap distance gradually decreases for all systems, confirming the densification of the magnetic particle network under compressive loading. At 15 N, the gap reductions of MRE-C, MRE-N, and MRE-P reach approximately 0.84%, 1.60%, and 2.18%, respectively. These results indicate that external compression promotes closer particle packing and enhances mechanical constraint within the polymer matrix. However, excessive compression may simultaneously restrict the mobility of magnetic particles, which is essential for reversible magnetic chain rearrangement under an applied magnetic field.

To further elucidate the effect of precompression on the magnetic-field-induced normal-force response, current-sweep measurements were performed from 0 to 5 A under precompression of 1, 3, 5, 7, 9, and 15 N at a frequency of 1 Hz and a strain amplitude of 0.1%, as shown in Figure 4B. Under the lowest precompression of 1 N, MRE-C and MRE-N exhibit comparatively smooth and continuous increases in normal force with increasing current, suggesting stable field-induced particle-chain formation and largely reversible microstructural rearrangement. MRE-P, by contrast, displays a more irregular response, including a localized decrease at intermediate currents, which may be associated with the structural defects and voids observed in its microstructure (Figure 3e,f). Such heterogeneities can disrupt stress transfer and promote nonuniform particle rearrangement, thereby reducing the stability of the evolving magnetic network. At 3 N, all formulations exhibit a more pronounced initial reduction in normal force followed by a rapid increase at higher currents, producing a characteristic U-shaped response. Abrupt changes near the upper end of the current range further suggest the onset of localized structural instability under the combined effects of magnetic loading and mechanical confinement. As the precompression increases to 5–15 N, discontinuous jumps and irregular fluctuations become progressively more prominent across all three MREs, particularly at intermediate and high currents. These features indicate that excessive compression restricts the relative motion of carbonyl iron particles and polymer chains, thereby hindering the continuous reconstruction of field-induced particle structures and promoting intermittent network rearrangement.

These results reveal a competing effect of precompression on the magneto-mechanical response of MREs. Such behavior is consistent with previous studies showing that pre-strain and compression can markedly influence the stiffness, MR response, and field-induced normal force of MREs [24,25]. In this work, moderate compression enhances particle contact, load transfer, and zero-field load-bearing capacity, whereas excessive mechanical confinement suppresses the microstructural mobility required for reversible magnetic-chain reorganization, resulting in increasingly unstable normal-force evolution. Among the investigated conditions, a precompression of 1 N provides the most stable and readily interpretable current-dependent response while preserving clear magnetic-field sensitivity, particularly for MRE-C and MRE-N. This condition was therefore selected to establish a reproducible loading state and to ensure reliable characterization of the intrinsic viscoelastic and magneto-responsive behavior of the MREs in subsequent experiments.

#### 3.2.2. Strain-Dependent Mechanical Stability

As shown in Figure 4C (Top), all three MREs exhibit a typical Payne effect, characterized by an initial strain-independent plateau followed by a pronounced decrease in storage modulus (G′) with increasing strain amplitude from 0.001% to 1%. This strain-softening behavior is consistent with previous reports on the magnetic-field-enhanced Payne effect in MREs, which has been associated with the progressive disruption and reorganization of particle networks under increasing deformation [26]. In the present systems, this behavior can be attributed to the progressive disruption, slippage, and rearrangement of magnetically induced particle chains and filler–matrix interactions under large deformation, leading to the transition from a stable linear viscoelastic region (LVR) to nonlinear viscoelastic behavior. The critical strains (γ_c_), determined by a 10% reduction in G′ relative to the initial plateau modulus, are 0.054%, 0.119%, and 0.080% for MRE-C, MRE-N, and MRE-P, respectively. At these critical points, the corresponding reductions in G′ are 11.5%, 9.9%, and 10.6%, confirming the validity of the selected criterion for identifying the boundary of the linear viscoelastic region. Meanwhile, the overall Payne effect amplitudes reach 75.33%, 37.90%, and 57.09%, respectively, indicating that MRE-N possesses the widest LVR and the strongest resistance to strain-induced softening. This enhanced strain stability is attributed to the regulating effect of naphthenic oil on the balance between polymer chain mobility and magnetic particle network confinement. Unlike simple plasticization, naphthenic oil facilitates particle rearrangement during vulcanization while maintaining effective interfacial constraints, thereby stabilizing the magnetic particle network and delaying its destruction during dynamic deformation. Consequently, MRE-N demonstrates a favorable balance between matrix flexibility and magnetic network integrity. This balance is reflected by its larger critical strain and lower Payne effect amplitude, suggesting a higher tolerance against strain-induced structural evolution during large-amplitude oscillatory deformation.

#### 3.2.3. Frequency-Dependent Viscoelastic Behavior

Frequency-dependent mechanical behavior was further investigated to elucidate the influence of plasticizers on the dynamic load-bearing capability and molecular relaxation behavior of MREs. As shown in Figure 4C (Bottom), the storage modulus (G′) of all MREs continuously increases with increasing frequency from low frequency to 100 Hz, indicating that rapid dynamic excitation restricts the relaxation of rubber molecular chains and enhances the dynamic coupling between carbonyl iron particles and the polymer matrix. The relative increases in G′ for MRE-C, MRE-N, and MRE-P reach 96.16%, 155.63%, and 183.49%, respectively. Although MRE-P exhibits the highest frequency-induced G′ increase, its overall modulus remains the lowest, indicating that the enhancement mainly originates from restricted chain relaxation and strengthened particle–matrix interactions instead of effective magnetic network strengthening. In contrast, MRE-N maintains the highest G′ throughout the entire frequency range, with its minimum and maximum G′ values being approximately 1.49 and 1.24 times those of MRE-P, respectively. This superior dynamic stiffness is attributed to the optimized balance between polymer chain mobility and magnetic network confinement provided by naphthenic oil. The enhanced chain mobility facilitates stress relaxation and dynamic response, while the preserved magnetic network integrity ensures effective load transfer under high-frequency excitation. Therefore, naphthenic oil enables a synergistic improvement in dynamic stiffness and structural stability by regulating the coupling between polymer relaxation behavior and magnetic particle network evolution.

Beyond elastic energy storage, G″ and tan δ are critical parameters for evaluating the viscoelastic dissipation behavior of MREs. G″ represents the dissipative energy associated with molecular friction, particle–matrix interfacial interactions, and magnetic network rearrangement during cyclic deformation, while tan δ describes the balance between viscous loss and elastic energy storage. As shown in Figure 4D (Top), the G″ of all MREs increases progressively with increasing frequency, indicating that high-frequency excitation restricts polymer chain relaxation and enhances molecular friction, particle–matrix interfacial interactions, and dynamic adjustment of magnetic structures, thereby promoting mechanical energy dissipation. Among the three systems, MRE-N maintains the highest G″ over the entire frequency range, increasing from approximately 0.009 MPa at low frequency to 0.062 MPa at 100 Hz, corresponding to an increase of approximately 589%. In comparison, MRE-C and MRE-P exhibit more pronounced relative increases in G″, increasing from approximately 0.005 and 0.003 MPa to 0.043 and 0.046 MPa, respectively, corresponding to increases of approximately 760% and 1433%. However, the large relative enhancement observed in these two systems mainly arises from their low initial dissipation levels and enhanced polymer chain mobility under dynamic excitation, rather than from a more stable magnetic network structure. Therefore, a higher G″ increase does not necessarily indicate superior dynamic performance, and the overall dissipative behavior of MREs should be evaluated by considering the synergistic interplay among viscous dissipation, elastic energy storage, and magnetic network stability. The frequency dependence of tan δ further reveals the differences in viscoelastic dissipation mechanisms among the three plasticizer systems. As shown in Figure 4D (Bottom), in the low-frequency region (0–20 Hz), all MREs exhibit relatively stable tan δ values, corresponding to the slow relaxation of polymer chains and limited interfacial friction under prolonged deformation periods. The initial tan δ of MRE-C, MRE-N, and MRE-P are approximately 0.100, 0.113, and 0.095, respectively, indicating that MRE-N exhibits a higher initial viscous response, whereas MRE-P shows a relatively lower contribution from viscous dissipation at low frequencies. As the frequency increases, the shortened deformation time restricts molecular chain relaxation and enhances the delayed response of polymer chains and particle–matrix interfaces, resulting in increased internal friction and viscoelastic loss. At 100 Hz, the tan δ of MRE-C, MRE-N, and MRE-P increases to 0.467, 0.317, and 0.523, corresponding to relative increases of 367%, 181%, and 451%, respectively. Although MRE-P exhibits the highest tan δ value at high frequency, its relatively low G′ suggests that the enhanced viscous response mainly originates from increased polymer chain mobility and particle–matrix interfacial sliding rather than effective dissipation supported by a stable magnetic network. In contrast, MRE-N maintains the lowest frequency sensitivity of tan δ while simultaneously exhibiting the highest G′ and G″, indicating a more favorable balance between elastic energy storage and viscous dissipation. These results demonstrate that the dynamic performance of MREs cannot be optimized by maximizing a single dissipation-related parameter. Instead, it requires a synergistic regulation of elastic support, viscous loss, and magnetic network stability.

#### 3.2.4. Magnetic-Field-Dependent Viscoelastic Response

The magnetically tunable mechanical response of MREs was evaluated through current sweep measurements, as shown in Figure 4E. With increasing applied current from 0 to 5 A, all MREs exhibit a continuous increase in storage modulus (G′), demonstrating that the enhanced magnetic field promotes the magnetization of carbonyl iron particles and facilitates the development of stronger magnetic chain structures within the polymer matrix. In the low-field region (0–2 A), MRE-N shows the most pronounced modulus enhancement, with G′ increasing from approximately 0.194 to 0.213 MPa (ΔG′ ≈ 0.019 MPa), exceeding the corresponding increases in MRE-C (0.009 MPa) and MRE-P (0.006 MPa). This behavior indicates that the naphthenic oil-regulated network achieves an optimized balance between particle mobility and matrix confinement, enabling efficient magnetic-field-driven particle rearrangement while maintaining structural stability of the magnetic network. At 5 A, the G′ values of MRE-C, MRE-N, and MRE-P reach approximately 0.196, 0.244, and 0.178 MPa, corresponding to increases of 24.1%, 25.8%, and 30.9%, respectively. Although MRE-P exhibits the highest relative magnetorheological enhancement, this is mainly attributed to its lower initial modulus rather than the formation of a more robust magnetic reinforcement network. In contrast, MRE-N maintains the highest G′ throughout the entire magnetic field range, with its maximum modulus exceeding those of MRE-C and MRE-P by approximately 24.5% and 37.1%, respectively, demonstrating superior magnetic stiffness regulation capability.

To further elucidate the magnetic-field-dependent dynamic dissipation behavior of MREs, the evolution of loss modulus (G″) and loss factor (tan δ) under varying magnetic fields was systematically investigated for the three plasticizer-dependent systems. As shown in Figure 4E (Center), all MREs exhibit magnetic-field-dependent variations in G″, although their response characteristics differ significantly. MRE-N maintains the highest G″ over the entire current range, increasing from approximately 0.022 MPa at 0 A to 0.027 MPa at 5 A, indicating enhanced field-induced dissipation behavior. In comparison, MRE-P increases from approximately 0.018 to 0.024 MPa, whereas MRE-C remains nearly unchanged around 0.019 MPa, suggesting a weaker modulation of dissipative response under applied magnetic fields. These differences originate from the distinct balance between polymer matrix flexibility, particle mobility, and magnetic network confinement. Previous studies have shown that compounding additives can modify matrix rheology and mechanical constraints, thereby affecting magnetic-particle mobility and field-induced rearrangement [22,27,28,29]. Consistent with these findings, plasticization-induced changes in matrix mobility and crosslink-related constraints have also been reported to strongly influence particle rearrangement and damping behavior in MREs [22,30]. Accordingly, the balance between particle mobility and matrix confinement is critical to the field-dependent viscoelastic response of MREs and varies with plasticizer type. The naphthenic oil-modified matrix provides sufficient chain mobility for carbonyl iron particles to migrate and rearrange under magnetic fields while preserving effective interfacial confinement, thereby promoting stable magnetic chain adjustment and continuous dissipation pathways. Although MRE-P exhibits a relatively larger increase in G″, its lower G′ indicates that the enhanced dissipation mainly arises from increased chain mobility and particle–matrix interfacial friction rather than a stable magnetic network. Conversely, the stronger matrix constraint in MRE-C restricts particle migration and magnetic chain rearrangement, leading to a slight decrease in G″ to approximately 0.017 MPa at 1.5–2 A. With further magnetic field enhancement, progressive magnetic chain adjustment restores interfacial dynamic interactions, resulting in the recovery of G″ toward its initial value. The variation in tan δ further reveals the balance between viscous dissipation and elastic energy storage under magnetic fields. MRE-P exhibits the highest tan δ values throughout the current sweep (approximately 0.128–0.133), indicating a stronger viscous contribution. However, considering its relatively low G′, the increased tan δ mainly originates from enhanced polymer chain mobility and interfacial sliding rather than effective dissipation supported by a stable magnetic network. For MRE-C, tan δ decreases from approximately 0.120 to 0.097 with increasing current, suggesting that magnetic-field-induced magnetic chain formation gradually increases elastic energy storage and reduces the relative contribution of viscous loss. In contrast, MRE-N maintains a relatively stable tan δ value (~0.111) while simultaneously exhibiting the highest G′ and G″, indicating a more favorable balance between magnetic stiffness enhancement and dynamic dissipation.

Combined with the SEM observations in Figure 3c,d, the superior magneto-viscoelastic performance of MRE-N can be attributed to its more continuous, compact, and uniformly distributed carbonyl iron particle chains, which provide efficient pathways for stress transfer and magnetic network evolution. Although paraffin enhances polymer chain mobility and facilitates particle migration, excessive matrix softening reduces the confinement capability required for maintaining stable magnetic chains. Conversely, the stronger constraint effect in the coumarone-based system limits particle rearrangement and magnetic network evolution. Therefore, the magnetic-field-dependent dynamic behavior of MREs is governed by the synergistic interplay among polymer chain mobility, particle migration ability, and magnetic network stability. By optimizing these structural factors, the naphthenic oil-based MRE achieves a favorable combination of magnetic stiffness regulation, stable dissipation response, and tunable magneto-viscoelastic behavior, providing valuable design insights for high-performance magnetically responsive elastomer composites.

Overall, the present results reveal distinct performance trade-offs among the three plasticized MREs. MRE-C retains relatively high absolute stiffness but exhibits greater strain-induced softening, whereas MRE-P provides stronger relative field tunability at the expense of absolute stiffness. MRE-N provides a more balanced combination of stiffness, strain stability, energy dissipation, and magnetic-field responsiveness, although its relative MR response is not the highest. Similar trade-offs have been reported for other plasticized MREs. Epoxidized palm oil has been shown to improve particle–matrix compatibility and MR response relative to naphthenic and light mineral oils [31], while different petroleum-based oil systems exhibit property-specific advantages in curing, microstructure, thermal stability, and magnetic characteristics [20]. Silicone-oil plasticization can substantially enhance the relative MR response through matrix softening, but this benefit is accompanied by reductions in zero-field stiffness and tensile strength [22]. These comparisons indicate that the effectiveness of a plasticizer should not be judged by MR response alone; rather, its suitability depends on the required balance between mechanical stability and field tunability.

### 3.3. Plasticizer-Dependent Vulcanization Characteristics and Crosslinking State

#### 3.3.1. Vulcanization Characteristics and Kinetic Behavior

The vulcanization process is a critical step in the formation of magnetorheological elastomers (MREs), during which the crosslinked network structure is established and ultimately determines the final crosslink density and mechanical performance of the materials. In this work, the effects of different plasticizers, including coumarone resin, naphthenic oil, and paraffin, on the vulcanization characteristics and kinetic behavior of MREs were systematically investigated.

**Figure 5:**
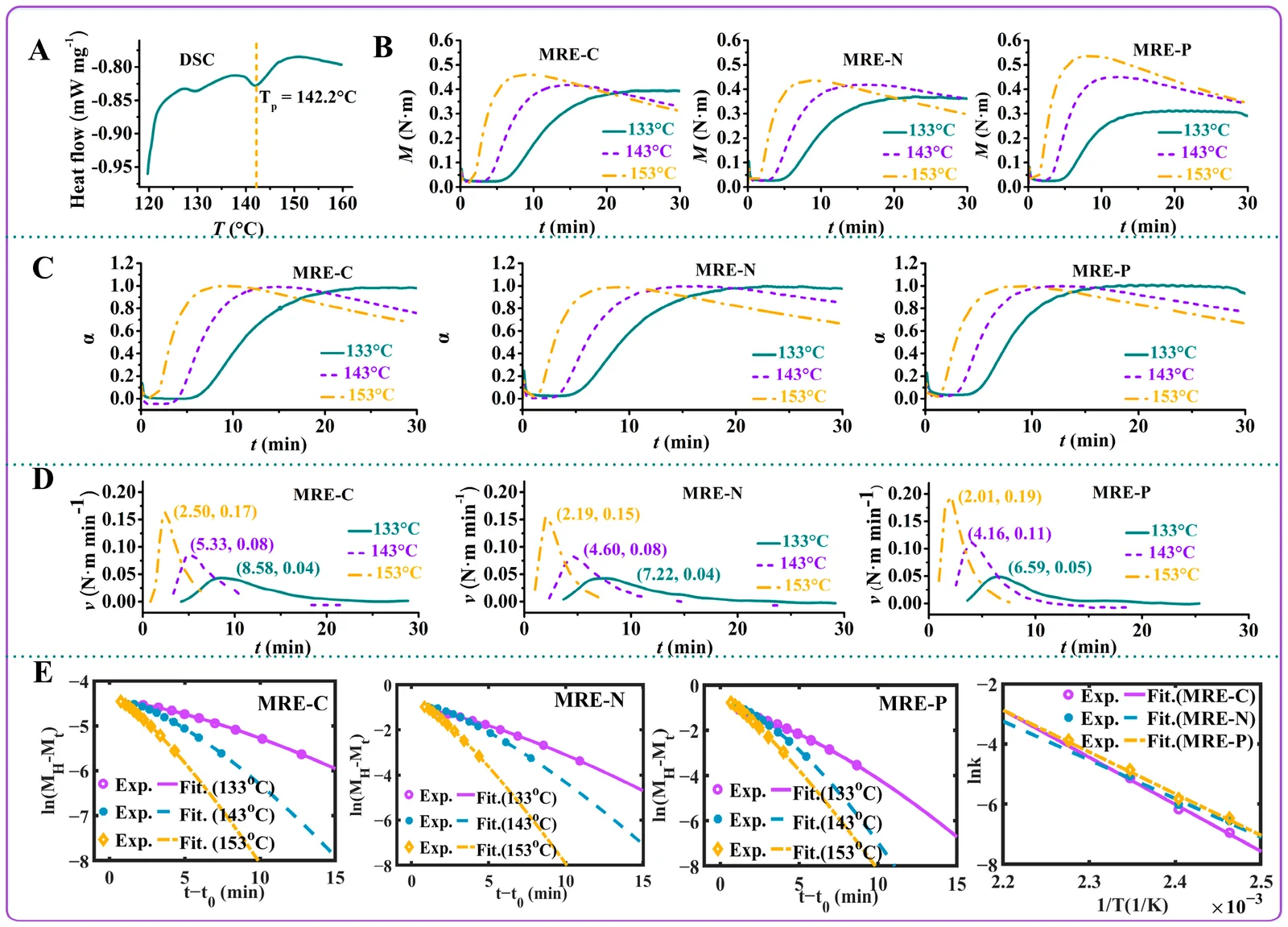
Vulcanization characteristics and kinetic behavior of MREs with different plasticizers. (**A**) DSC curve. (**B**) Curing torque–time curves. (**C**) Evolution of relative crosslinking degree. (**D**) Vulcanization-rate profiles. (**E**) Kinetic-model fitting and Arrhenius analysis results.

The curing behavior was first analyzed by differential scanning calorimetry (DSC823E, Mettler-Toledo, Greifensee, Switzerland)). The test was performed under a nitrogen atmosphere with a flow rate of 20 mL·min^−1^. The sample mass was 6.95 mg, and the heating rate was 10 °C·min^−1^ over a temperature range of 120–160 °C, with a data acquisition frequency of 50 points per second. As shown in Figure 5A, the DSC curve exhibits a pronounced exothermic peak at 142.2 °C, corresponding to the onset of the vulcanization reaction. With increasing temperature, the exothermic signal gradually diminishes and eventually disappears, indicating completion of curing. Although DSC provides valuable thermodynamic insight into the reaction onset and heat flow evolution, the relatively weak exothermic response inherent to MRE vulcanization restricts its capability to resolve the full curing dynamics and quantify the reaction state with high accuracy.

To further characterize the curing process, a rotorless rheometer was used to monitor torque evolution of MREs. Figure 5B shows the vulcanization (torque–time) behavior of MREs containing different plasticizers, including coumarone resin (MRE-C), naphthenic oil (MRE-N), and paraffin (MRE-P), under curing temperatures of 133, 143, and 153 °C, which were selected based on the reported suitable curing temperature of natural rubber and the temperature fluctuations commonly encountered during practical vulcanization. Table 2 summarizes the corresponding vulcanization parameters for quantitative comparison. In all systems, the torque (M) increases with curing time, reflecting the progressive formation of sulfur crosslinked networks in the rubber matrix, followed by a slower growth stage and a subsequent plateau or post-peak decline, which is attributed to the competition between crosslink formation and thermally induced network rearrangement or reversion. At 133 °C, the coumarone resin- and naphthenic oil-based MREs exhibit a typical three-stage curing behavior consisting of an induction period, a main vulcanization stage, and a plateau stage, whereas the paraffin-based system additionally shows an over-curing stage, indicating partial network degradation. With increasing temperature (133 → 143 → 153 °C), all systems exhibit an earlier onset of torque rise, a higher maximum torque, and a faster curing rate, confirming accelerated vulcanization kinetics. Meanwhile, more pronounced post-peak torque decay appears at higher temperatures, indicating intensified reversion behavior. Consistently, Table 2 shows that the minimum torque (ML) remains nearly constant at 0.02–0.03 N·m, indicating weak temperature dependence of the initial compound viscosity. In contrast, the maximum torque (MH) increases with temperature, reaching the highest value in the paraffin system (0.57 N·m), followed by coumarone resin (0.46 N·m) and naphthenic oil (0.44 N·m), suggesting enhanced but composition-dependent network development. Meanwhile, both scorch time (TS1) and optimum curing time (T_90_) decrease significantly with increasing temperature. Specifically, T_90_ decreases from 1038, 920, and 771 s at 133 °C to 337, 315, and 317 s at 153 °C for coumarone resin, naphthenic oil, and paraffin systems, respectively. At 133 °C, the paraffin-based MRE exhibits the shortest T_90_, indicating a relatively faster attainment of the optimum curing state under mild thermal conditions. However, with increasing temperature, the curing kinetics of all systems are significantly accelerated, and the naphthenic oil-based MRE becomes dominant in curing efficiency at elevated temperature, exhibiting the lowest T_90_ (315 s at 153 °C). This transition suggests that the kinetic advantage of paraffin at lower temperatures is gradually overtaken by the superior mobility-mediated network formation efficiency of the naphthenic oil system under higher thermal activation. Overall, it has been demonstrated that curing temperature primarily governs vulcanization kinetics, while plasticizer type critically regulates the balance between crosslink formation efficiency and network stability.

Building on the previously discussed torque evolution behavior, the evolution of the crosslinking state was further evaluated using a torque-normalized relative crosslinking degree *α*, following established rheometer-based cure analysis [32], as expressed in Equation (3).

(3) $$\alpha =\left(M_{t}-M_{0}\right)/\left(M_{H}-M_{0}\right)$$

where *M*_t_ is the torque at any vulcanization time *t*, *M*_0_ is the torque at *t* = 0, and *M*_H_ is the maximum torque.

Figure 5C shows the evolution of the relative crosslinking degree, α, for MRE-C, MRE-N, and MRE-P cured at 133, 143, and 153 °C. After a short induction period, all systems exhibited a sigmoidal increase in α, followed by either a stable plateau or a post-maximum decline. Increasing the curing temperature markedly accelerated network formation, reducing the time required to reach α = 0.90 from 17.77, 15.33, and 12.27 min at 133 °C to 5.62, 5.38, and 5.28 min at 153 °C for MRE-C, MRE-N, and MRE-P, respectively. This acceleration was accompanied by increasingly pronounced post-cure reversion. At 133 °C, MRE-C and MRE-N retained 99.2% and 97.7% of their maximum crosslinking degrees at 30 min, respectively, indicating relatively stable network development under mild curing conditions. MRE-P showed a more evident rise–maximum–decline profile and retained only 92.3% of its maximum value, suggesting greater susceptibility to network rearrangement even at the lowest temperature. At 143 °C, post-maximum decay became apparent in all three systems. MRE-N exhibited the highest retention of 84.8%, compared with 76.7% for MRE-C and 77.0% for MRE-P, demonstrating superior resistance to reversion under intermediate-temperature curing. At 153 °C, all systems reached their maxima within approximately 8.5–9.2 min and subsequently decreased to similar final values of 0.664–0.668, corresponding to losses of approximately 33% from their respective maxima. The convergence of the three curves at this temperature indicates that severe thermal reversion increasingly dominates over plasticizer-dependent stabilization. Overall, the plasticizer type governs both curing kinetics and post-cure network retention. Paraffin promotes relatively rapid crosslink development but provides limited resistance to reversion, coumarone resin produces slower yet highly stable network formation at 133 °C, and naphthenic oil offers the most favorable balance between curing rate and network retention, particularly at 143 °C. These results demonstrate that the apparent crosslinking evolution of MREs is controlled by the competition between sulfur-network formation and thermally activated network rearrangement, with the relative contribution of each process strongly dependent on both curing temperature and plasticizer type.

In addition to the crosslinking state, the vulcanization rate (*v*) is also a critical kinetic factor that must be considered during practical MRE fabrication. It is characterized by the vulcanization rate constant (k) and is quantified from the time-dependent variation in rheometer torque during thermal vulcanization using established rheometer-based kinetic treatments [33], as expressed in Equation (4).

(4) $$v=-\frac{d(M_{H}-M_{t})}{dt}=k{\left(M_{H}-M_{t}\right)}^{n}$$

Figure 5D shows the curing-rate profiles of MRE-C, MRE-N, and MRE-P at 133, 143, and 153 °C. All formulations exhibit a well-defined single maximum, reflecting an initial acceleration of sulfur crosslink formation followed by progressive deceleration as active crosslinking species are consumed and the developing network increasingly restricts molecular mobility. Increasing the curing temperature substantially intensified and compressed this kinetic response, shifting the rate maximum to shorter times while markedly increasing its magnitude. For MRE-C, the peak curing rate increased from 0.04 N·m·min^−1^ at 8.58 min and 133 °C to 0.08 N·m·min^−1^ at 5.33 min and 143 °C and further to 0.17 N·m·min^−1^ at 2.50 min and 153 °C. Similar trends were observed for MRE-N, with peak coordinates of 7.22 min and 0.04 N·m·min^−1^, 4.60 min and 0.08 N·m·min^−1^, and 2.19 min and 0.15 N·m·min^−1^, respectively. MRE-P consistently exhibited the earliest and highest rate maxima, reaching 0.05 N·m·min^−1^ at 6.59 min, 0.11 N·m·min^−1^ at 4.16 min, and 0.19 N·m·min^−1^ at 2.01 min as the temperature increased from 133 to 153 °C. These results demonstrate that paraffin promotes the most rapid and concentrated crosslinking response, whereas coumarone resin produces a slower and broader curing process, with naphthenic oil showing intermediate behavior. The increasingly sharp rate peaks at elevated temperatures indicate a progressively narrower effective curing window, particularly for MRE-P, implying greater sensitivity to curing time and an increased risk of overcuring. Overall, the curing-rate evolution is jointly governed by temperature and plasticizer type, which regulate polymer-chain mobility, transport of active sulfur species, and the temporal development of the crosslinked network.

To further determine the vulcanization rate constant, Equation (4) was integrated to obtain the kinetic expression shown in Equation (5) [33].

(5) $$\ln(M_{H}-M_{t})=-k{\left(t-t_{0}\right)}^{n}+B$$

where *t*_0_ represents the curing time corresponding to the minimum torque, and *n* denotes the reaction order.

Using Equation (5), the complete curing profiles of MRE-C, MRE-N, and MRE-P recorded after the minimum-torque point at 133, 143, and 153 °C were fitted to determine their apparent vulcanization kinetic parameters. The linearized fitting results are presented in Figure 5E, and the corresponding kinetic and Arrhenius parameters were summarized in Table 3. The consistently high coefficients, with R^2^ values ranging from 0.9997 to 0.9999, confirm that the adopted model accurately describes the overall evolution of sulfur-crosslinked network formation over the investigated curing interval. The fitted apparent reaction orders range from 1.222 to 1.545 and are non-integers for all formulations, indicating that the obtained values represent global kinetic orders associated with a multistep vulcanization process rather than a single elementary reaction. This process likely involves the generation and consumption of active sulfur species, formation and rearrangement of sulfur crosslinks, polymer-chain reorganization, and the progressive restriction of molecular mobility as the network develops. The fitted rate constant k increases markedly with curing temperature. As the temperature rose from 133 to 153 °C, k it increases from 0.05761 to 0.353 for MRE-C, from 0.08498 to 0.377 for MRE-N, and from 0.09245 to 0.4616 for MRE-P. At each temperature, the rate constants followed the order MRE-P > MRE-N > MRE-C, consistent with the earlier and higher curing-rate maxima observed in Figure 5D. These results demonstrate that temperature provides the principal driving force for accelerating sulfur-network formation, whereas plasticizer type modulates the relative kinetic response of the rubber matrix.

To quantify the thermal activation behavior underlying the fitted vulcanization kinetics, the temperature dependence of the vulcanization rate constant *k* was further analyzed using the Arrhenius equation in Equation (6) [33].

(6) $$\ln k=\ln A-\frac{E}{RT}$$

where *A*, *E*, *R*, and *T* represent the pre-exponential factor, apparent activation energy, gas constant, and absolute curing temperature, respectively.

The Arrhenius plots exhibit an approximately linear relationship between ln*k* and 1/T, with coefficients of determination of 0.9902, 0.9724, and 0.9842 for MRE-C, MRE-N, and MRE-P, respectively. These results confirm that vulcanization is predominantly governed by thermally activated kinetics over the investigated temperature range. The corresponding apparent activation energies were 130.11, 106.83, and 115.40 kJ mol^−1^, respectively. The lower activation energy of MRE-N indicates that naphthenic oil reduces the apparent kinetic barrier to network formation, most likely by promoting polymer-chain mobility and improving the accessibility of active sulfur species and reactive sites. Notably, MRE-P exhibits the highest rate constants despite having a higher activation energy than MRE-N. This behavior can be attributed to its larger pre-exponential contribution, as reflected by the higher ln*A* value of 27.66 compared with 25.03 for MRE-N, indicating that the observed vulcanization rate is jointly governed by the activation barrier and the frequency of effective reactive events.

Taken together with the curing-rate profiles and the evolution of relative crosslinking degree, the kinetic results indicate that a higher vulcanization rate does not necessarily lead to a more stable crosslinked network. Paraffin enables MRE-P to exhibit the fastest curing response; however, its sharp rate maximum, narrow effective processing window, and pronounced post-peak decrease in relative crosslinking degree reveal a greater susceptibility to overcuring and network reversion. By contrast, the coumarone-resin-containing system shows the slowest vulcanization kinetics and the highest apparent activation energy yet maintains comparatively stable network development under relatively mild curing conditions. The naphthenic-oil-containing system provides a more favorable balance among kinetic accessibility, curing efficiency, and post-cure network retention. Overall, curing temperature primarily governs the rate of sulfur-crosslink formation, whereas plasticizer type controls the broader vulcanization response by modulating polymer-chain mobility, the transport and accessibility of reactive sulfur species, crosslink-network development, and the stability of the evolving network structure.

#### 3.3.2. Crosslink Density

**Figure 6:**
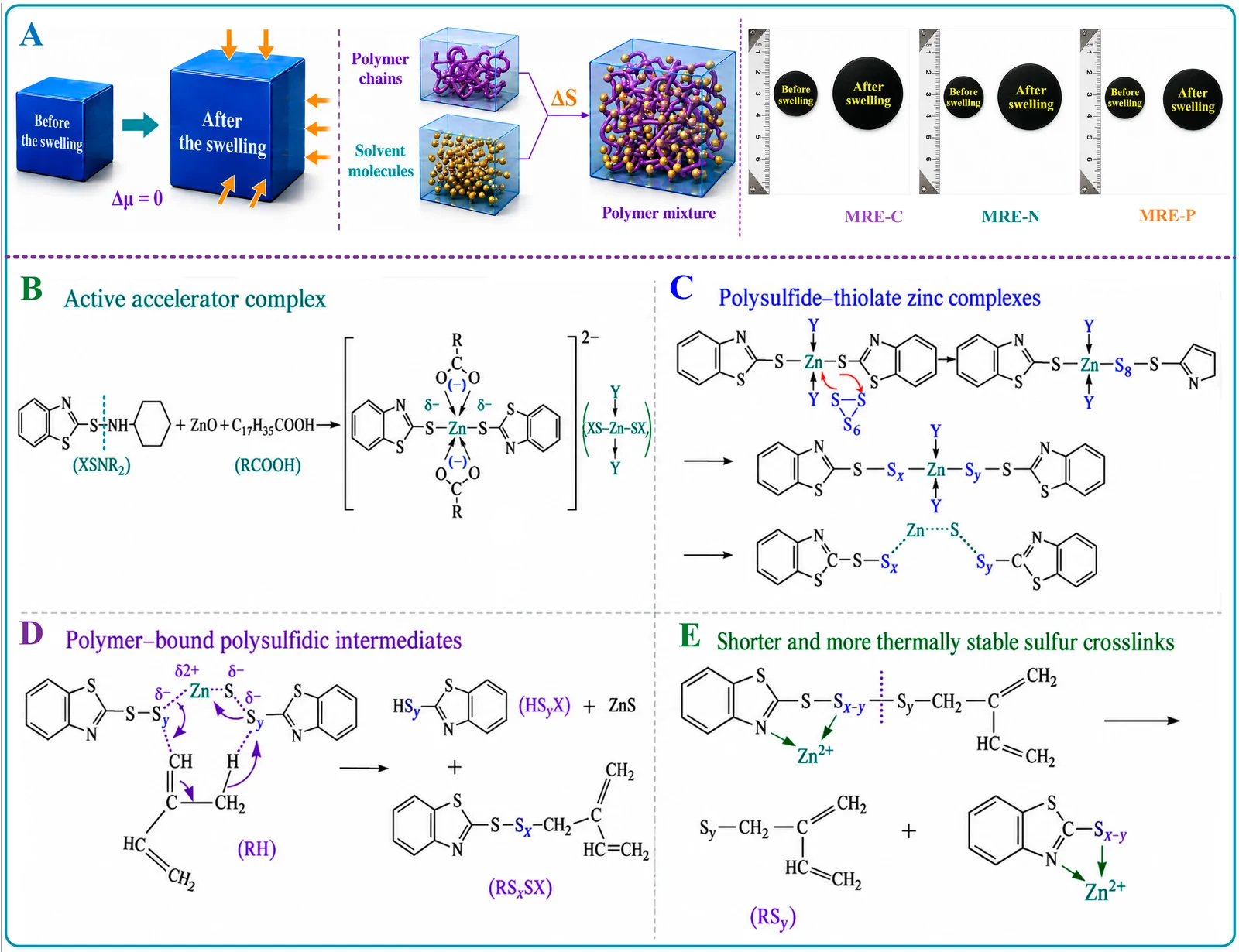
Swelling behavior and sulfur-crosslink formation mechanism of MREs. (**A**) Swelling process and representative morphologies. (**B**–**E**) Literature-based schematic of the accelerated sulfur-vulcanization pathway (Adapted from [40,41,42]).

The crosslink density of magnetorheological elastomers (MREs) was quantitatively determined using the equilibrium swelling method, which is based on the thermodynamic balance between solvent diffusion into the polymer network and the elastic retraction force of the crosslinked structure. As illustrated in Figure 6A, swelling occurs when solvent molecules penetrate the three-dimensional rubber network, leading to chain expansion and volumetric increase. This process continues until swelling equilibrium is reached, at which point the chemical potential of the solvent inside the polymer equals that of the external solvent (Δ*μ*_1_ = 0), indicating a balance between osmotic pressure and network elasticity [34].

The crosslink density ($v_{e}$) was calculated using the Flory–Rehner relationship [35], as shown in Equation (7). A higher crosslink density indicates a higher degree of vulcanization in MREs.

(7) $$v_{e}=-\frac{\;\ln\left(1-\varphi_{2}\right)+\varphi_{2}+\chi_{1}\varphi_{2}^{2}}{\rho_{2}V_{m,1}\varphi_{2}^{\frac{1}{3}}}$$

According to the derived crosslink density equation, the calculation of MREs containing coumarone resin, naphthenic oil, and paraffin requires determining the polymer–solvent interaction parameter ($\chi_{1}$); the solvent molar volume ($V_{m,1}$); and the polymer volume fraction ($\varphi_{2}$), which is obtained from the measured masses of the samples in the swollen and dried states. The corresponding relationship is given in Equation (8).

(8) $$\varphi_{2}=\frac{\frac{m_{3}}{\rho_{2}}}{\frac{m_{2}-m_{3}}{\rho_{1}}+\frac{m_{3}}{\rho_{2}}}$$

where $\rho_{1}$ is the density of the solvent, and *m*_2_ and *m*_3_ denote the masses of the MRE in the swollen state and after drying, respectively. The densities of all materials were measured using an AR-300G densimeter manufactured by Hangzhou Jinmai Instrument Co., Ltd., Hangzhou, Zhejiang, China.

Based on the crosslink-density equation derived above and the associated parameters, the crosslink density of the MREs formulated with the three plasticizers were tested as below. Pre-weighed samples (*m*_1_) were immersed in 50 mL of cyclohexane (*ρ*_1_ = 0.778 g·cm^−3^, $V_{m,1}$ = 108.2 cm^3^·mol^−1^) in sealed amber glass bottles and maintained at 40 °C for 48 h. During the swelling process, samples were periodically removed, and excess surface solvent was gently blotted using filter paper. The specimens were immediately transferred to sealed weighing containers for mass measurement and then returned to the solvent. This procedure was repeated until the mass variation between two consecutive measurements was less than 0.01 g, indicating that swelling equilibrium had been reached. The equilibrium swollen mass was recorded as *m*_2_. After reaching equilibrium, the samples were removed and dried in a vacuum oven at 50 °C to constant weight, and the final dry mass was recorded as *m*_3_.

As illustrated by the representative swelling and deswelling morphologies in Figure 6A, all MREs containing different plasticizers undergo pronounced volumetric expansion upon solvent uptake, reflecting the diffusion of cyclohexane into the three-dimensional crosslinked rubber network. Thermodynamically, swelling is governed by the reduction in the Gibbs free energy of polymer–solvent mixing, to which the increase in configurational entropy (ΔS) contributes substantially. As solvent penetrates the rubber matrix, the resulting osmotic driving force promotes network expansion, whereas deformation of the crosslinked chains generates an opposing elastic retractive force. Equilibrium swelling is established when these two contributions become balanced, such that no further net solvent uptake occurs.

Since natural rubber consists predominantly of cis-1,4-polyisoprene and cyclohexane was used as the swelling solvent, the polymer–solvent interaction parameter (χ_1_) was selected based on reported data for the polyisoprene/cyclohexane system [36]. Considering the polymer volume fractions obtained in this study and the literature data at 45 °C, which is close to the swelling temperature of 40 °C, χ_1_ = 0.30 was adopted for the crosslink-density calculation using Equation (10). The measured densities of MRE-C, MRE-N, and MRE-P were 1.959, 1.975, and 2.005 g cm^−3^, respectively. Substitution of these values, together with the interaction parameter and equilibrium swelling data, into the crosslink-density relationship yielded apparent crosslink density of 2.07 × 10^−4^, 2.22 × 10^−4^, and 1.88 × 10^−4^ mol·cm^−3^ for MRE-C, MRE-N, and MRE-P, respectively. MRE-N exhibits the highest crosslink density, indicating the formation of a more densely connected rubber network, whereas MRE-P shows the lowest value, consistent with comparatively limited network connectivity. These results demonstrate that plasticizer chemistry plays a decisive role in regulating the final crosslink density by altering polymer-chain mobility, the accessibility of reactive species, and the development of the sulfur-crosslinked network during vulcanization.

#### 3.3.3. Model-Based Analysis of Crosslink Network Development and Degradation

Building on the preceding results, plasticizer type was found to markedly influence the vulcanization kinetics and crosslink density of the MREs. However, these macroscopic parameters alone provide limited insight into the kinetic processes underlying crosslink-network development and degradation. MRE-N was therefore selected for further analysis because it exhibited the lowest apparent activation energy, the highest crosslink density, and the most favorable overall magneto-viscoelastic performance among the investigated formulations. An established Coran-type reaction framework was subsequently employed to track the evolution of key reactive species and examine the competing processes associated with crosslink formation, network development, and degradation [37,38,39].

As illustrated in Figure 6B–E, the vulcanization process proceeds through four partially overlapping stages, representing a continuous evolution from sulfur activation to network maturation. The accelerator employed in this work is the sulfenamide-type N-cyclohexyl-2-benzothiazole sulfenamide (CZ), which exhibits delayed-action characteristics due to its long induction period and near-neutral reactivity. In the presence of ZnO and stearic acid, CZ forms an active accelerator complex (Figure 6B) with significantly enhanced reactivity, thereby facilitating sulfur transfer and initiating the vulcanization process. These complexes subsequently react with S_8_ via ring-opening to generate polysulfidic sulfurating species, serving as primary crosslink precursors. Concurrently, nucleophilic interaction at S–Zn coordination sites leads to the formation of polysulfide–thiolate zinc complexes (Figure 6C), where Zn–S coordination stabilizes reactive intermediates and governs subsequent crosslink formation pathways. In the second stage, the active sulfurating species react with the α-H adjacent to the C=C bonds in cis-1,4-polyisoprene units ((–C_5_H_8_–)_n_) of natural rubber, where allylic hydrogens are readily activated. This reaction leads to the formation of polymer-bound polysulfidic intermediates with sulfur–accelerator moieties grafted onto the rubber backbone (Figure 6D). The third stage is dominated by interchain crosslink formation through coupling reactions between activated intermediates. Once the concentration of crosslink precursors reaches a critical level, rapid network construction occurs. In the presence of Zn^2+^, chelation with polysulfidic side groups stabilizes labile bonds and redirects S–S bond scission pathways, leading to the formation of shorter and more thermally stable sulfur crosslinks (Figure 6E). Concurrently, radical species generated during bond rearrangement couple to form initial polysulfidic crosslink structures, while benzothiazole-terminated radicals react with rubber chains to regenerate active side groups, which further act as new crosslink precursors and promote continuous network growth, thereby increasing crosslink density. In the final stage, the crosslinked network undergoes further maturation, where polysulfidic linkages progressively convert into shorter disulfidic and monosulfidic bonds under the action of active accelerator species. Simultaneously, bond cleavage and reformation of sulfur crosslinks establish a dynamic equilibrium between chain shortening and network reconstruction. This process competes with thermally induced degradation, including backbone scission, structural rearrangement, and cyclization, ultimately determining the final network architecture and its thermal stability.

To quantitatively analyze the competition between crosslink formation and network degradation, the established kinetic framework proposed by Coran and subsequently extended by Ding and Leonov was adopted [40,41,42]. The reaction scheme accounts for precursor formation, crosslink formation, competing side reactions, and crosslink reversion, as is shown in Equation (9).

(9) $$\left\{\begin{array}{l} A\overset{k_{1}}{\to }B\overset{k_{2}}{\to }B^{*}\overset{k_{3}}{\to }\alpha V_{u}\\ A+B^{*}\overset{k_{4}}{\to }\beta B\\ B^{*}\overset{k_{5}}{\to }\gamma D\\ V_{u}\overset{k_{6}}{\to }\gamma D \end{array}\right.$$

where *A* denotes the active sulfurating agent formed from the reaction of accelerator, activator, and sulfur. *B* represents the crosslink precursor, a sulfur- and accelerator-containing polysulfidic species that initiates crosslinking upon reaching a critical concentration. B* is the activated form of B, in which bond cleavage generates shorter sulfur linkages and free radicals. *V*_u_ denotes the vulcanized product. *α*, *β*, and *γ* are stoichiometric coefficients, *k* is the reaction rate constant, and *D* represents inert by-products.

Based on the established model, the time-dependent crosslink density after the induction period can be expressed as Equation (10) [42].

(10) $$c_{V_{u}}(t_{j})=\frac{k_{2}c_{A0}(e^{-k_{6}t_{j}}-e^{-k_{2}t_{j}})}{\left(k_{2}-k_{6}\right)\left(1+M\right)}$$

where *c_V_*_u_ (*t*_j_) denotes the concentration of the crosslinking-related species at the effective reaction time *t*_j_; *c*_*A*0_ is the initial concentration of component A; *k*_2_ and *k*_6_ are the rate constants associated with the formation and subsequent consumption of *V*_u_, respectively; *M* is a dimensionless parameter related to the composition or stoichiometric ratio of the reaction system; *t_i_* denotes the induction time; and *t_j_* = *t* − *t_i_* represents the effective crosslinking time after the induction period. At *t* = *t_m_* (where *t* denotes the total reaction time measured from the beginning of the reaction and *t_m_* denotes the curing time corresponding to the maximum crosslink density), the degree of crosslinking reaches its maximum, and Equation (11) is obtained.

(11) $$\frac{dc_{V_{u}}}{dt_{j}}=0$$

Solving Equation (11) yields Equation (12).

(12) $$t_{jm}=t_{m}-t_{i}=\frac{\ln\left(k_{2}/k_{6}\right)}{k_{2}-k_{6}}$$

By normalizing the instantaneous crosslink density to its maximum value, the vulcanization state S used for kinetic fitting is obtained, as shown in Equation (13).

(13) $$S=\frac{c_{V_{u}}(t)}{c_{V_{u}}(m)}={\left(\frac{k_{2}}{k_{6}}\right)}^{\frac{k_{6}}{k_{2}-k_{6}}}\cdot \frac{e^{-k_{6}(t-t_{i})}-e^{-k_{2}(t-t_{i})}}{1-k_{6}/k_{2}}$$

where *k*_2_ is associated with the formation of activated crosslink precursors and subsequent network formation, with a larger value indicating faster crosslink formation. In contrast, *k*_6_ is associated with the reversion reaction, where a larger value indicates more severe thermal degradation.

The parameters *k*_2_ and *k*_6_ in Equation (13) were determined by nonlinear least-squares fitting, and the corresponding kinetic results for the naphthenic oil-plasticized MRE (MRE-N) at different curing temperatures are summarized in Table 4. Both *k*_2_ and *k*_6_ increase with increasing curing temperature, indicating that elevated temperature accelerates both crosslink formation and the competing reversion process. Throughout the investigated temperature range, *k*_2_ remains substantially higher than *k*_6_, suggesting that crosslink formation is kinetically dominant over network degradation. Accordingly, the ratio *k*_2_/*k*_6_, used here as an indicator of the relative dominance of network formation over degradation, increases from 9.910 to 16.208 as the curing temperature rises from 133 to 153 °C. Meanwhile, the predicted *t_m_* decreases markedly from 1418.48 to 516.77 s as the curing temperature increases, indicating a substantial acceleration in reaching the maximum crosslink density. This temperature-dependent trend is consistent with the previously reported kinetic behavior of sulfur vulcanization [39,43]. The consistently high R^2^ values (>0.997) further indicate good agreement between the kinetic model and the experimental data. Therefore, for naphthenic oil-plasticized MREs, the curing temperature should be carefully optimized to achieve a desirable balance between rapid network formation and reversion suppression.

### 3.4. Molecular-Level Analysis of Sulfur-Crosslink Stability

#### 3.4.1. Equilibrium Structures of Representative Sulfur Bridges

The kinetic analysis in Section 3.3.3 indicates that the sulfur-crosslinked network develops through competing processes of crosslink formation, maturation, and degradation. However, the kinetic model does not directly resolve the structural characteristics of individual sulfur linkages. Previous studies have shown that higher-rank polysulfidic crosslinks can undergo desulfuration and rearrangement during network maturation, giving rise to shorter sulfur linkages, among which monosulfidic and disulfidic crosslinks represent important low-sulfur-rank structures [44,45]. These two configurations were therefore selected as representative sulfur-bridge motifs for molecular-level analysis.

**Figure 7:**
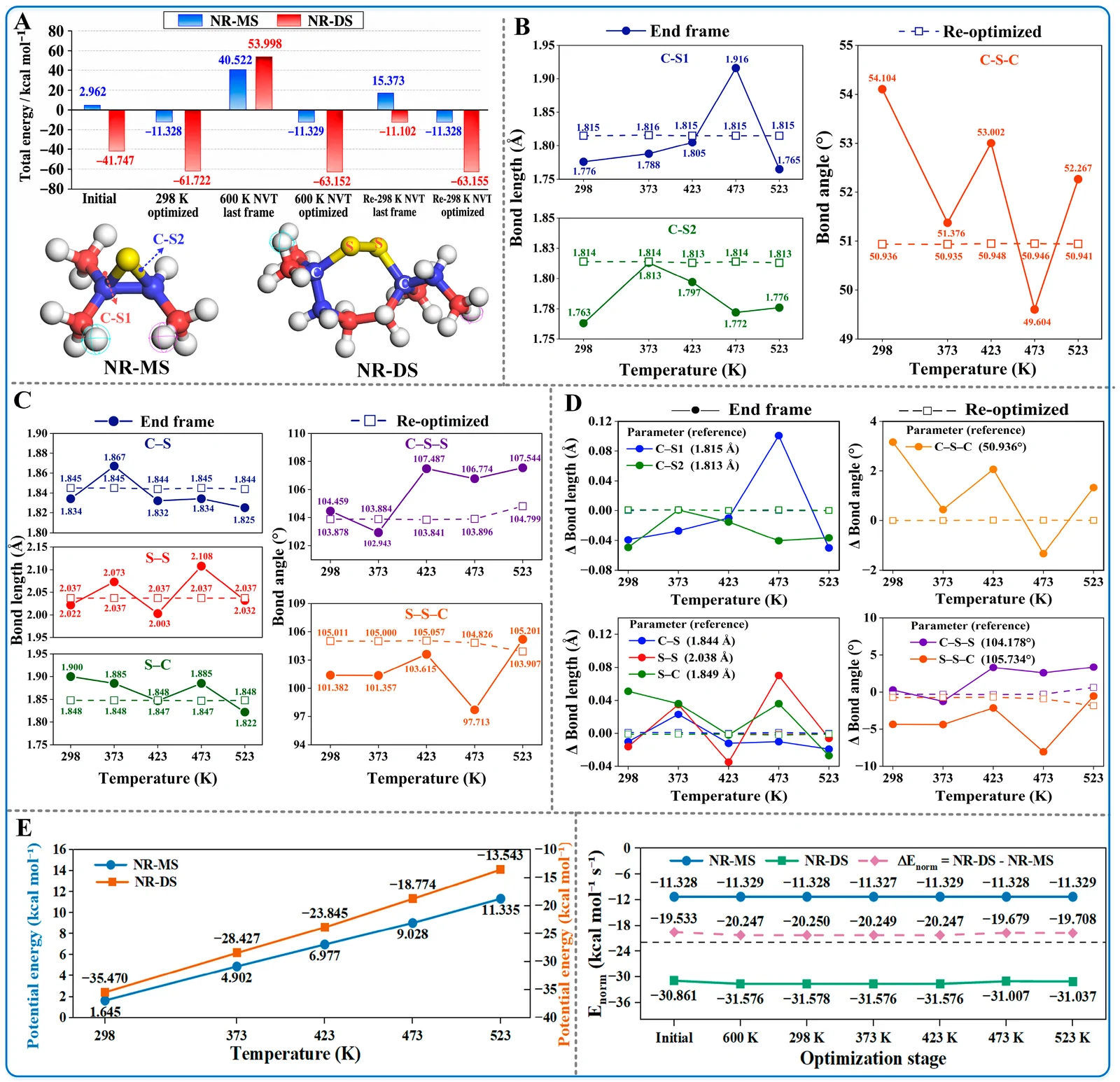
Structural and energetic responses of NR-MS and NR-DS to thermal loading. (**A**) Energy evolution and optimized structures. (**B**) NR-MS bond lengths and angle. (**C**) NR-DS bond lengths and angles. (**D**) Structural deviations from the 298 K reference. (**E**) Potential and sulfur-normalized energies.

To establish equilibrated reference structures for subsequent temperature-dependent analysis, the NR-MS and NR-DS models described in Section 2.3 were subjected to sequential structural relaxation and equilibration. The initial configurations were first geometry-optimized at 298 K to eliminate unfavorable local interactions and obtain locally relaxed structures, providing a consistent basis for subsequent energetic and geometric comparisons between the two sulfur-bridge configurations. As shown in Figure 7A, the total energies of NR-MS and NR-DS decreased from 2.962 to −11.328 kcal mol^−1^ and from −41.747 to −61.722 kcal mol^−1^, corresponding to energy reductions of 14.290 and 19.975 kcal mol^−1^, respectively. Because the two models differ in chemical composition, these absolute energies were used to evaluate the extent of stabilization within each model rather than to directly compare their thermodynamic stability. NR-MS exhibited only minor geometric relaxation. The C–S1 and C–S2 bond lengths changed from 1.808 and 1.815 Å to 1.815 and 1.813 Å, respectively, while the C–S–C angle increased slightly from 50.176° to 50.936°. Thus, the absolute changes in the two C–S bond lengths were only 0.007 and 0.002 Å, and the angular variation was limited to 0.760°, indicating that the initial NR-MS configuration was already close to a local energy minimum. Geometry optimization therefore induced only slight bond-length equalization and angular relaxation, without substantial conformational reconstruction. By contrast, NR-DS underwent a markedly greater and more coordinated structural rearrangement. Its C–S, S–S, and S–C bond lengths changed from 1.807, 2.072, and 1.806 Å to 1.844, 2.038, and 1.849 Å, respectively, while the C–S–S and S–S–C angles decreased from 104.838° and 110.100° to 104.178° and 105.734°. The corresponding absolute bond-length changes ranged from 0.034 to 0.043 Å, substantially exceeding the range of 0.002–0.007 Å observed for NR-MS. The mean absolute bond-length change in NR-DS was approximately 0.038 Å, about 8.4 times that in NR-MS, while its maximum angular adjustment reached 4.366°, compared with only 0.760° for NR-MS. These quantitative differences provide direct evidence that the disulfidic bridge undergoes considerably greater local relaxation than the monosulfidic bridge. Notably, the contraction of the central S–S bond occurred concurrently with elongation of the two terminal C–S bonds and asymmetric angular adjustment of the C–S–S–C linkage. Stabilization of NR-DS therefore arises not from uniform contraction of the crosslink but from the coordinated redistribution of bond-length and angular distortions, which relieves local geometric strain and lowers the configurational energy. Overall, the contrasting relaxation modes demonstrate that sulfur-bridge topology governs the manner in which local strain is accommodated. The compact and geometrically constrained C–S–C linkage enables NR-MS to largely preserve its initial geometry, whereas the more conformationally flexible C–S–S–C linkage allows NR-DS to dissipate local strain through internal structural redistribution. The optimized configurations consequently provide well-defined reference states for subsequent analyses of thermal perturbation, structural recovery, and energy evolution.

Following geometry optimization at 298 K, the NR-MS and NR-DS models were subjected to an NVT simulation at 600 K to promote thermally activated conformational sampling and assess the capacity of their sulfur bridges to accommodate and recover from thermal perturbation. The final trajectory frames were subsequently re-optimized, allowing transient thermal distortions to be distinguished from persistent structural rearrangements. The energy profiles in Figure 7A reveal a pronounced increase in the energies of the 600 K final configurations, reaching 40.522 kcal mol^−1^ for NR-MS and 53.998 kcal mol^−1^ for NR-DS. Subsequent geometry optimization reduced the energies to −11.329 and −63.152 kcal mol^−1^, respectively. The post-annealing energy of NR-MS was essentially identical to its pre-annealing value of −11.328 kcal mol^−1^, whereas NR-DS reached an energy 1.430 kcal mol^−1^ lower than its initially optimized value of −61.722 kcal mol^−1^. These energetic responses indicate that thermal treatment induces predominantly reversible deformation in NR-MS, while the greater conformational freedom of NR-DS enables exploration of a broader configurational space and access to a more favorable local minimum. The corresponding geometric evolution provides direct support for this interpretation. In NR-MS, the two C–S bonds changed from 1.815 and 1.813 Å before thermal treatment to 1.903 and 1.820 Å at 600 K, while the C–S–C angle decreased from 50.936° to 48.847°. This strongly asymmetric bond extension indicates that thermal strain was concentrated primarily along one C–S linkage within the compact monosulfidic bridge. After re-optimization, however, the C–S bond lengths recovered to 1.815 and 1.814 Å and the C–S–C angle returned to 50.940°, demonstrating nearly complete restoration of the pre-annealing geometry. The monosulfidic bridge therefore responds to thermal excitation through localized but highly reversible deformation, consistent with the strong geometric constraints imposed by its compact C–S–C topology. In contrast, NR-DS exhibited a more distributed and cooperative response. During the 600 K simulation, the C–S, S–S, and S–C bond lengths changed from 1.844, 2.038, and 1.849 Å to 1.819, 2.011, and 1.830 Å, respectively, while the C–S–S and S–S–C angles decreased from 104.178° and 105.734° to 102.407° and 100.103°. The simultaneous adjustment of all three bond lengths, together with the pronounced 5.631° decrease in the S–S–C angle, shows that thermal strain was redistributed across multiple internal degrees of freedom within the C–S–S–C linkage. Following re-optimization, the bond lengths returned to 1.845, 2.037, and 1.848 Å, while the corresponding angles recovered to 103.891° and 105.015°, confirming that the disulfidic bridge remained structurally intact despite its greater thermal flexibility. Collectively, these results demonstrate that sulfur-bridge topology governs both the pathway of thermal strain accommodation and the extent of configurational recovery. The geometrically constrained monosulfidic bridge preserves structural integrity through localized and almost completely reversible distortion, whereas the more flexible disulfidic bridge dissipates thermal stress through coordinated internal reorganization and accesses a slightly more stable relaxed configuration.

Upon completion of the 600 K annealing and subsequent re-optimization, both NR-MS and NR-DS were re-equilibrated under the NVT ensemble at 298 K to establish ambient-temperature reference configurations. As indicated by the energy profiles in Figure 7A, the final 298 K trajectory frames exhibited total energies of 15.373 kcal mol^−1^ for NR-MS and −11.102 kcal mol^−1^ for NR-DS, indicating that residual thermal distortions remained in the dynamically equilibrated structures. Subsequent geometry optimization reduced these energies to −11.328 and −63.155 kcal mol^−1^, corresponding to decreases of 26.701 and 52.053 kcal mol^−1^, respectively. These optimized values differed by only approximately 0.001 and 0.003 kcal mol^−1^ from those obtained after optimization of the preceding 600 K trajectories, confirming that both systems returned to essentially the same low-energy basins. The resulting equilibrated and optimized structures at 298 K were therefore employed as common reference configurations for the subsequent investigation of temperature-dependent structural evolution and recovery.

#### 3.4.2. Temperature-Dependent Structural Response

To examine the temperature-dependent structural response of the representative sulfur bridges, independent NVT simulations were performed at 373, 423, 473, and 523 K using the equilibrated and geometry-optimized structures at 298 K as common reference configurations. The final configuration obtained at each temperature was subsequently geometry-optimized to assess structural relaxation following thermal perturbation. Changes in characteristic bond lengths and bond angles before and after optimization were then compared with the corresponding 298 K reference values to quantify thermally induced geometric deviations and evaluate the recovery of the local sulfur-bridge structures.

As shown in Figure 7B, the thermal perturbation induces only limited variations in the local C–S–C cyclic structure of NR-MS. Across the overall temperature range, the C–S bond lengths in the NVT configurations fluctuate within 1.765–1.916 Å, with the largest deviation observed at 473 K, indicating transient bond stretching caused by enhanced thermal motion. Meanwhile, the C–S–C bond angle exhibits moderate variation between 49.604° and 54.104°. After geometry optimization, the C–S bond lengths consistently recovered to 1.813–1.816 Å, while the C–S–C angle remained stabilized at approximately 50.94°. These results demonstrate that the monosulfidic bridge primarily accommodates thermal perturbation through reversible local geometric adjustment while maintaining the intrinsic structural constraint of the compact C–S–C cyclic configuration. In comparison, NR-DS exhibits a more pronounced structural response during thermal loading due to the incorporation of an additional sulfur atom within the crosslink bridge, which introduces greater conformational freedom. As shown in Figure 7C, the thermally perturbed NR-DS configurations show significant variations in the S–S bridge and associated angular parameters. Specifically, the S–S bond length varied from 2.003 to 2.108 Å and reached its maximum elongation at 473 K, while the C–S–S and S–S–C bond angles fluctuate within 102.943–107.544° and 97.713–105.201°, respectively. These structural deviations indicate that the disulfidic bridge responds to thermal perturbation through coordinated adjustment of the S–S bond length and angular degrees of freedom. After geometry optimization, the S–S bond length is restored to approximately 2.037 Å, while the terminal C–S and S–C bonds remain within narrow ranges of 1.844–1.845 Å and 1.847–1.848 Å, respectively. Meanwhile, the C–S–S and S–S–C angles recover to stable ranges of approximately 103.8–104.8° and 103.9–105.1°, respectively. The recovery of these structural parameters confirms that NR-DS accommodates thermally induced deformation through reversible internal rearrangement rather than irreversible structural disruption. The deviation profiles in Figure 7D, calculated relative to the geometry-optimized 298 K reference structures, provide a quantitative comparison of the topology-dependent thermal response and subsequent recovery of the two sulfur bridges. For NR-MS, the deviations in the final NVT frames were dominated by asymmetric fluctuations of the two C–S bonds. The C–S1 bond exhibited the largest deviation of +0.101 Å at 473 K, whereas the C–S2 bond varied within −0.050 to 0 Å. The corresponding C–S–C angular deviation ranged from −1.332° to +3.168°. After re-optimization, however, the residual deviations were reduced to no more than 0.001 Å for the C–S bonds and 0.012° for the C–S–C angle, demonstrating nearly complete recovery of the compact monosulfidic geometry. In contrast, NR-DS displays a more distributed response involving both bond-length and angular degrees of freedom. In the final frames, the C–S, S–S, and S–C bond deviations ranged from −0.019 to +0.023 Å, −0.035 to +0.070 Å, and −0.027 to +0.051 Å, respectively. More pronounced variations occurred in the angular coordinates, with the C–S–S angle deviating by −1.235° to +3.366° and the S–S–C angle by −8.021° to −0.533°. Re-optimization restored all three bond lengths to within 0.002 Å of their reference values, although modest angular offsets remained, particularly for the S–S–C angle, whose residual deviation ranged from −1.827° to −0.677°. These results indicate that the monosulfidic bridge accommodates thermal loading primarily through localized and almost completely reversible deformation, whereas the disulfidic bridge redistributes strain cooperatively across the S–S bond and adjacent angular coordinates. Moreover, the nonmonotonic variation in the end-frame parameters with temperature argues against a simple progressive accumulation of local distortion over the investigated range. Instead, the data reveal temperature-dependent conformational fluctuations followed by substantial structural recovery, with NR-MS showing nearly complete geometric restoration and NR-DS retaining minor residual angular relaxation despite recovery of its bond-length framework.

The topology-dependent structural responses of NR-MS and NR-DS were further examined in relation to their energetic evolution under thermal perturbation. As shown in Figure 7E, the average potential energy of both models increases monotonically from 298 to 523 K, consistent with enhanced molecular motion and broader configurational sampling at elevated temperatures. For NR-MS, the average potential energy rose from 1.645 to 11.335 kcal mol^−1^, corresponding to an overall increase of 9.69 kcal mol^−1^. This comparatively moderate change is consistent with the restricted conformational response of the geometrically constrained monosulfidic bridge. By contrast, the average potential energy of NR-DS increases from −35.470 to −13.543 kcal mol^−1^, yielding a larger variation of approximately 21.93 kcal mol^−1^. The stronger energetic response of NR-DS agrees with its greater conformational freedom and the more extensive redistribution of strain across the S–S bond and adjacent angular degrees of freedom. Although NR-DS retains more negative potential-energy values than NR-MS throughout the investigated temperature range, the absolute energies of the two models should not be directly interpreted as measures of their relative thermodynamic stability because they differ in chemical composition and sulfur content.

Normalization of the optimized total energy by the number of sulfur atoms provides a composition-adjusted descriptor for comparing the energetic response associated with the two crosslink topologies. As shown in Figure 7E, the sulfur-normalized energy of NR-MS remains essentially invariant at approximately −11.33 kcal mol^−1^ S^−1^ throughout all optimization stages, with fluctuations of less than 0.002 kcal mol^−1^ S^−1^. This remarkable constancy indicates that the geometrically constrained C–S–C bridge repeatedly returned to nearly the same local energy basin after thermal perturbation. NR-DS exhibits similarly stable normalized energies of approximately −31.58 kcal mol^−1^ S^−1^ from the 600 K annealing stage through the 423 K treatment, followed by modest increases to −31.007 and −31.037 kcal mol^−1^ S^−1^ after exposure to 473 and 523 K, respectively. Correspondingly, the normalized energy difference remains close to −20.25 kcal mol^−1^ S^−1^ up to 423 K and narrowed slightly to −19.679 and −19.708 kcal mol^−1^ S^−1^ at 473 and 523 K. Within the adopted normalization framework, NR-DS consistently exhibits a more negative optimized energy per sulfur atom than NR-MS. The modest shifts observed after treatment at 473 and 523 K suggest slight changes in the relaxed conformation or local nonbonded interaction environment rather than disruption of the disulfidic bridge. When interpreted together with the structural-deviation analysis, these energetic trends reveal two distinct but complementary stabilization mechanisms. NR-MS preserves structural integrity through its compact and strongly constrained C–S–C linkage, which confines thermally induced deformation to a limited number of local coordinates and enables nearly complete recovery of both geometry and energy after optimization. By contrast, the additional S–S linkage in NR-DS introduces greater conformational freedom, allowing thermal strain to be redistributed cooperatively across the S–S bond and adjacent angular degrees of freedom. Although NR-DS exhibits larger instantaneous structural deviations, its characteristic bond-length framework is largely restored after optimization and its normalized energy remains within a narrow range. The residual angular relaxation and slight energy shifts at the two highest temperatures therefore indicate relaxation into closely related local minima rather than irreversible structural alteration. Sulfur-bridge topology consequently governs not only the magnitude of thermal deformation but also the pathway through which local strain is accommodated, redistributed, and released.

These configuration-dependent responses reveal distinct structural characteristics of monosulfidic and disulfidic bridges under thermal perturbation. Monosulfidic linkages exhibit stronger geometric constraint and a greater tendency to recover toward their reference configurations, whereas disulfidic linkages show greater conformational flexibility and accommodate thermally induced structural distortions through local rearrangement. These contrasting responses suggest that the relative contributions of geometrically constrained and conformationally flexible sulfur linkages may influence the balance between structural retention and adaptability in sulfur-crosslinked elastomer networks. Because the distribution of sulfur-crosslink types is sensitive to vulcanization formulation and curing conditions, controlling crosslink structure may provide a potential route for tailoring this balance. Overall, the present analysis establishes a configuration-dependent relationship between local sulfur-bridge geometry, energetic relaxation, and thermal response, providing molecular-level insight into the structural roles of representative sulfur crosslinks in vulcanized elastomers.

## 4. Conclusions

This study systematically compared natural rubber-based MREs formulated with coumarone resin (MRE-C), naphthenic oil (MRE-N), and paraffin (MRE-P) to clarify how plasticizer type influences microstructural characteristics, magneto-viscoelastic performance, vulcanization behavior, and crosslinking state. MRE-C was most susceptible to strain-induced softening, whereas MRE-P showed a high relative magnetic-field response but was limited by its low absolute stiffness. In contrast, MRE-N developed more continuous anisotropic particle-chain structures and achieved the most favorable overall balance, with a critical strain approximately 120% and 49% higher and a Payne-effect amplitude approximately 50% and 34% lower than those of MRE-C and MRE-P, respectively, indicating greater resistance to strain-induced structural disruption. MRE-N also maintained the highest storage and loss moduli over the investigated frequency range. Under magnetic excitation, although its G′ enhancement at 5 A was 5.1 percentage points lower than that of MRE-P, its absolute G′ was approximately 24.5% and 37.1% higher than those of MRE-C and MRE-P, respectively. Its G″ was likewise approximately 42.0% and 12.5% higher, with little change in tan δ, demonstrating that MRE-N retained strong magnetic-field responsiveness while maintaining higher stiffness and dissipative capability. The enhanced macroscopic performance of MRE-N was accompanied by more favorable vulcanization and crosslinking characteristics, as evidenced by apparent activation energies 17.9% and 7.4% lower and apparent crosslink densities 7.2% and 18.1% higher than those of MRE-C and MRE-P, respectively. Further analysis of the selected MRE-N using an established vulcanization kinetic model characterized the competition between crosslink formation and network degradation. Molecular simulations of representative mono- and disulfidic bridges revealed distinct responses to mechanical and thermal perturbations, with monosulfidic bridges showing stronger geometric constraint and structural recovery and disulfidic bridges exhibiting greater conformational flexibility. Overall, the experimental results link plasticizer-dependent vulcanization and crosslinking state with macroscopic magneto-viscoelastic performance, while the molecular simulations provide further structural insight into representative sulfur crosslinks. Among the formulations investigated, naphthenic oil provided the most favorable balance of structural stability, stiffness, energy dissipation, and magnetic-field responsiveness.

## Figures and Tables

**Table 1:** Chemical names and structures of accelerator CZ, antioxidant RD, and antioxidant 4010NA.

Additive	Chemical Name	Chemical Structure
Accelerator CZ	N-cyclohexyl-2-benzothiazolesulfenamide (CBS)	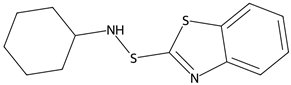
Antioxidant RD	Poly(2,2,4-trimethyl-1,2-dihydroquinoline) (TMQ)	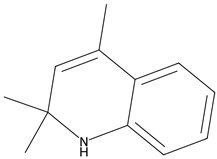
Antioxidant 4010NA	N-isopropyl-N′-phenyl-p-phenylenediamine (IPPD)	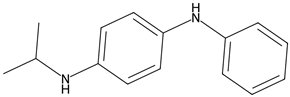

**Table 2:** Vulcanization parameters of MRE with different plasticizers at various temperatures.

Plasticizers	Curing Temperature (°C)	ML (N·m)	MH (N·m)	TS1 (s)	T_90_ (s)
Coumarone resin	133	0.02	0.4	527	1038
	143	0.02	0.42	311	602
	153	0.02	0.46	148	337
Naphthenic oil	133	0.03	0.37	455	920
	143	0.03	0.42	286	603
	153	0.02	0.44	136	315
Paraffin	133	0.02	0.31	420	771
	143	0.03	0.46	243	483
	153	0.03	0.57	119	317

**Table 3:** Kinetic fitting parameters of MREs with different plasticizers.

Plasticizers	T (°C)	k (1/N·m·min)	*n*	*B*	R^2^	E (kJ/mol)	lnA	$R_{1}^{2}$
Coumarone resin	133	0.05761	1.464	−0.8756	0.9998	130.11	31.56	0.9902
	143	0.1247	1.491	−0.7487	0.9999			
	153	0.353	1.324	−0.6762	0.9999			
Naphthenic oil	133	0.08498	1.391	−1.022	0.9998	106.83	25.03	0.9724
	143	0.1467	1.391	−0.7275	0.9998			
	153	0.377	1.286	−0.6547	0.9998			
Paraffin	133	0.09245	1.515	−1.117	0.9997	115.40	27.66	0.9842
	143	0.1766	1.545	−0.7565	0.9999			
	153	0.4616	1.222	−0.4718	0.9998			

**Table 4:** Vulcanization fitting parameters of MRE-N at different temperatures.

T (°C)	$k_{2}$ (s^−1^)	$k_{6}$ (s^−1^)	$k_{2}/k_{6}$	Predicted *t_m_* (s)	*R* ^2^
133	0.0026477	0.0002672	9.910	1418.48	0.999318
143	0.0042672	0.0003468	12.305	926.24	0.999182
153	0.0077965	0.0004810	16.208	516.77	0.997661

## Data Availability

The data presented in this study are available from the corresponding author upon reasonable request.
